# Genome sequencing of evolved aspergilli populations reveals robust genomes, transversions in *A. flavus*, and sexual aberrancy in non-homologous end-joining mutants

**DOI:** 10.1186/s12915-019-0702-0

**Published:** 2019-11-11

**Authors:** Isidro Álvarez-Escribano, Christoph Sasse, Jin Woo Bok, Hyunsoo Na, Mojgan Amirebrahimi, Anna Lipzen, Wendy Schackwitz, Joel Martin, Kerrie Barry, Gabriel Gutiérrez, Sara Cea-Sánchez, Ana T. Marcos, Igor V. Grigoriev, Nancy P. Keller, Gerhard H. Braus, David Cánovas

**Affiliations:** 1 Department of Genetics, Faculty of Biology, University of Seville, Seville, Spain; 2 Present Address: Instituto de Bioquímica Vegetal y Fotosíntesis, Consejo Superior de Investigaciones Científicas y Universidad de Sevilla, Seville, Spain; 3 Department of Molecular Microbiology and Genetics and Göttingen Center for Molecular Biosciences (GZMB), Georg-August-University, Göttingen, Germany; 4 Department of Medical Microbiology and Immunology, University of Wisconsin-Madison, Madison, WI USA; 5 US Department of Energy Joint Genome Institute, Walnut Creek, CA USA; 6 Present Address: Instituto para el Estudio de la Reproducción Humana (Inebir), Avda de la Cruz Roja 1, 41009 Sevilla, Spain; 7 Department of Plant and Microbial Biology, University of California Berkeley, Berkeley, CA USA; 8 Department of Bacteriology, University of Wisconsin-Madison, Madison, WI USA

**Keywords:** *Aspergillus*, Aflatoxin, Mutation accumulating lines, Genome stability, Non-homologous end-joining, *ku70*

## Abstract

**Background:**

*Aspergillus* spp. comprises a very diverse group of lower eukaryotes with a high relevance for industrial applications and clinical implications. These multinucleate species are often cultured for many generations in the laboratory, which can unknowingly propagate hidden genetic mutations. To assess the likelihood of such events, we studied the genome stability of aspergilli by using a combination of mutation accumulation (MA) lines and whole genome sequencing.

**Results:**

We sequenced the whole genomes of 30 asexual and 10 sexual MA lines of three *Aspergillus* species (*A. flavus*, *A. fumigatus* and *A. nidulans*) and estimated that each MA line accumulated mutations for over 4000 mitoses during asexual cycles. We estimated mutation rates of 4.2 × 10^−11^ (*A. flavus*), 1.1 × 10^−11^ (*A. fumigatus*) and 4.1 × 10^−11^ (*A. nidulans*) per site per mitosis, suggesting that the genomes are very robust. Unexpectedly, we found a very high rate of GC → TA transversions only in *A. flavus*. In parallel, 30 asexual lines of the non-homologous end-joining (NHEJ) mutants of the three species were also allowed to accumulate mutations for the same number of mitoses. Sequencing of these NHEJ MA lines gave an estimated mutation rate of 5.1 × 10^−11^ (*A. flavus*), 2.2 × 10^−11^ (*A. fumigatus*) and 4.5 × 10^−11^ (*A. nidulans*) per base per mitosis, which is slightly higher than in the wild-type strains and some ~ 5–6 times lower than in the yeasts. Additionally, in *A. nidulans*, we found a NHEJ-dependent interference of the sexual cycle that is independent of the accumulation of mutations.

**Conclusions:**

We present for the first time direct counts of the mutation rate of filamentous fungal species and find that *Aspergillus* genomes are very robust. Deletion of the NHEJ machinery results in a slight increase in the mutation rate, but at a rate we suggest is still safe to use for biotechnology purposes. Unexpectedly, we found GC→TA transversions predominated only in the species *A. flavus*, which could be generated by the hepatocarcinogen secondary metabolite aflatoxin. Lastly, a strong effect of the NHEJ mutation in self-crossing was observed and an increase in the mutations of the asexual lines was quantified.

## Introduction

The taxon of filamentous fungi comprises organisms of great importance for human daily life, as friends or foes. As friends, we take advantage of the capabilities of filamentous fungi to produce enzymes and compounds of interest to humans, such as pharmaceuticals. Some of these enzymes have proved to be highly useful in the production of bio-energy [1, 2] and fungal natural products impactful in many applications such as the antifungal echinocandins [3]. Within the filamentous fungi group, aspergilli are widely used for biotechnological applications in industry. In particular, they have become model organisms to study different aspects of the bio-energy production, such as the production of cellulolytic enzymes useful for bioethanol production [4–6], the carbon-regulation of these enzymes [4, 7] and natural product synthesis as drug sources [3, 8, 9]. These studies rely on two important aspects of the biology of aspergilli: their fantastic features for fermentation purposes and the genetic amenability of the genus. The availibilty of many genome sequences of *Aspergillus* species makes this group of fungi very useful for comparative genomics [10–12]. As foes, some *Aspergillus* species are pathogens of humans, animals and/or plants, or appear as dangerous contaminants of crops before or after the harvest. They are capable of producing an ample diversity of secondary metabolites, such as the carcinogen aflatoxin [13–16].

**Fig. 1:**
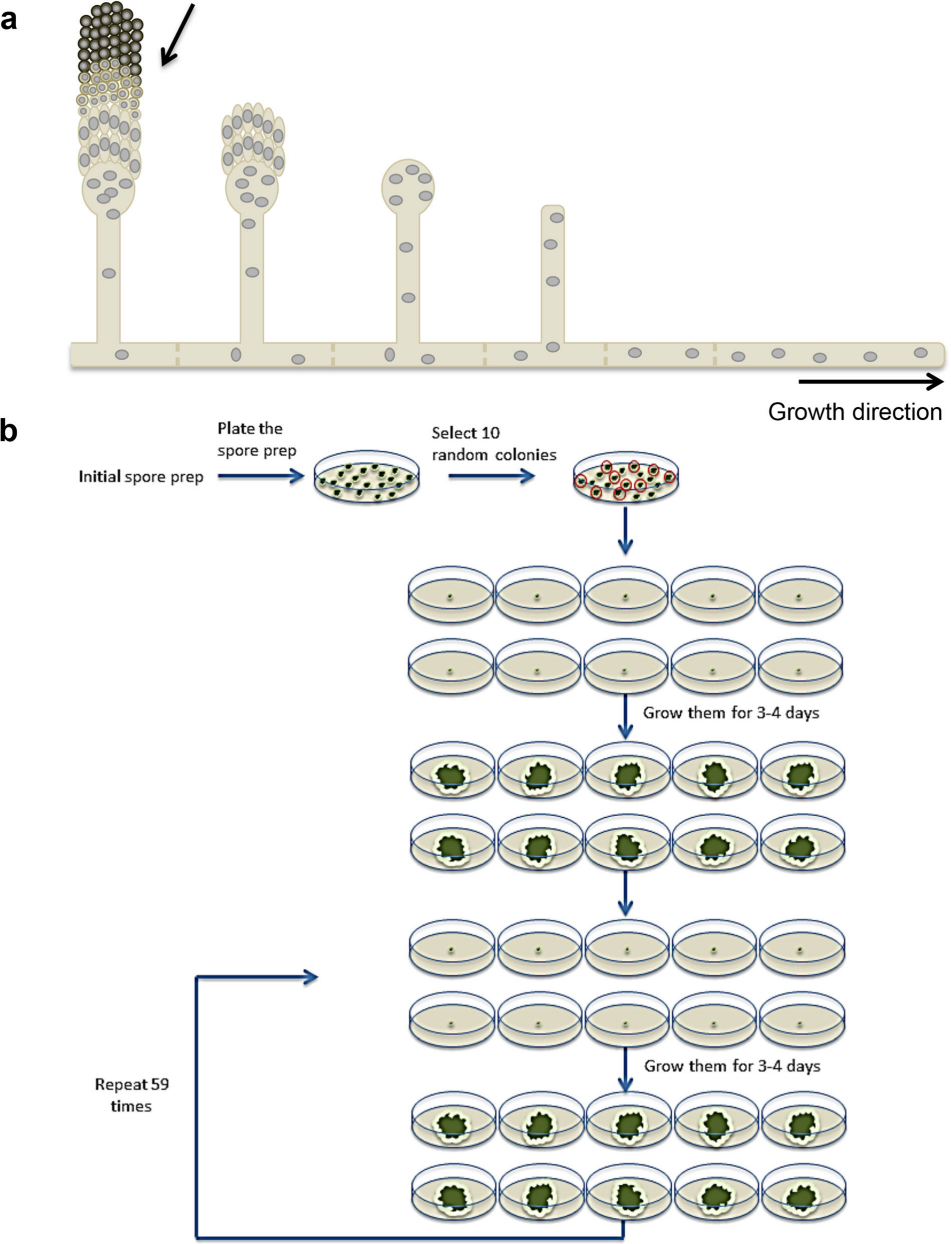
Diagram depicting the design of the evolution experiment in laboratory. **a** Diagram depicting the morphological characteristics of filamentous fungi. Growth occurred by apical extension at the tip. The older parts of the filament are separated in cell compartments by incomplete septa. The cell compartments are multinucleated. After the correct signals are perceived, the stalk arises from the filament. A morphogenetic program will control the development of the conidiophore, which harbours the uninucleated conidia. **b** A spore prep of each strain was diluted and plated to obtain isolated colonies. Ten random colonies showing normal wild-type characteristics were selected to establish each MA line and inoculated in an individual plate containing complete media. Fungal colonies were allowed to grow for 3 days at 37 °C (30 °C for *A. flavus*). One plug was taken from the conidiating edge of each plate, conidia were resuspended in 1000 μl of tween buffer and 10 μl were used to inoculate a new fresh plate. The procedure was repeated alternatively every 3 and 4 days (twice a week) 60 times to reach approximately 4032 mitosis

However, the evolution of aspergilli is still understudied. One of the main problems studying the evolution of fitness in filamentous fungi is the concept of an individual [17] (see Fig. 1a for a diagram for the morphological structure of a filamentous fungus). Thus, most evolutionary studies in filamentous fungi involved the comparison between different species or extremely laborious phenotypic analysis in combination with genetic techniques, e.g. RFLP analysis [18]. Some previous studies have shown that filamentous fungal evolution can be assessed experimentally under laboratory conditions [19, 20]. Using mutation accumulating (MA) lines for 40 generations, the base pair mutation rate in *Aspergillus nidulans* was estimated to be 2.26 × 10^−10^ using the classical Bateman-Mukai method [21]. The recent availability of the genome sequences can boost the study of filamentous fungal evolution in a laboratory experiment to a level never experienced before. So far, evolution experiments in filamentous fungi have considered growth rates as a measurement of fitness. However, variations in growth rate could be the result of a physiological adaptation to the established growth conditions rather than the consequence of genome evolution.

The stability of genomes highly depends on the lifestyle and the complexity of each organism. Whereas viruses have very dynamic genomes, eukaryotic genomes are highly stable and eukaryotes do not cope well with insults to the genome. Yet cells are continuously exposed to DNA damaging agents. Therefore, the stability of the genome relies on a complex molecular machinery that repairs the damage. One such example of DNA repair machinery is the non-homologous end-joining (NHEJ), which joins two DNA molecules that had suffered double-strand breaks (DSBs) [22–24]. Thus, a doubt arose with the discovery of the NHEJ genes in filamentous fungi and the use of the NHEJ deletions as genetic tools [25]. The underlying fundamental question behind the doubt is what is the role of the NHEJ system in the stability of an eukaryotic genome. Although this is a fundamental question in biology, the knowledge has direct consequences on biotechnology with the use of NHEJ mutants to generate new fungal strains with desired genetic modifications. The fungal homologues of the *KU70* and *KU80* required for NHEJ were first identified in *Neurospora crassa* [25]. Following the spectacular results of *N. crassa ∆ku70* strains as genetic tools, NHEJ mutants have been constructed in many other fungal species (for some examples, see [26–31]). *N. crassa* NHEJ mutants are more sensitive to bleomycin, a compound producing double-strand DNA breaks [25], although an *A. nidulans* NHEJ mutant did not appear to show increase sensitivity to this drug [32]. However, there is an additional body of data generated in fungi that suggests that the KU70/KU80 heterodimer plays additional roles. For example, *N. crassa* and *Aspergillus fumigatus* mutants, but not *A. nidulans*, are more sensitive to agents producing point mutations [25, 33, 34], which were presumed to get repaired using different mechanisms. They are telomere-associated proteins contributing to the formation of heterochromatin in the telomeres [35]. In *A. nidulans*, NkuA^KU70^ appears to regulate telomere position effects [36]. Both *ku70* and *ku80* homologues were found to be overexpressed during asexual reproduction (conidiation) in a histone acetyltransferase mutant, suggesting that they are involved in the maintenance of genome stability in conidia, DNA repair or the responses to DNA stress [37]. In haploid cells, as *Aspergillus* species normally propagate, DNA repair by homologous recombination (HR) of double-strand breaks is only possible in S and G2 phases of the cell cycle, when a sister chromatid is available. Therefore, the repair of double-strand breaks is expected to be more error-prone in a haploid because of the higher likelihood of using NHEJ repair.

As these additional roles of the KU70/KU80 heterodimer on fungal biology and potentially DNA stability have emerged, it is prudent for the academic community and industry to determine if the NHEJ mutants display concerning genome instability properties. A stable genome is especially important for biotechnology applications, where much time and money is spent to optimize processes and a small change in yield can have large financial consequences. In addition to insights on *Aspergillus* genome evolution and stability, we expect our results will be widely applicable to other fungi where the use of NHEJ mutants is expanding [28, 38, 39]. Therefore, and considering that strains lacking KU70/KU80 are commonplace in biotechnology, we decided to test the global consequences of such mutants for the stability of the genome. We studied the genome stability of aspergilli by using a combination of MA lines and whole genome sequencing (WGS). We found that *Aspergillus* genomes are very robust and the deletion of the NHEJ machinery results in a slight increase in the mutation rate, but at a rate, which we postulate, is still safe to use for biotechnology purposes.

## Results

### MA lines, sequencing and identification of the mutations

Three different aspergilli species have been selected based on their different characteristics and relevance in the aspergilli community: *A. nidulans*, *A. flavus* and *A. fumigatus*. All species were already sequenced before this work started, and they have genome sizes ranging between 29.4 and 36.8 Mb [11, 40]. Ten independent MA lines for each strain (wild type and *∆ku70* of each species) were set up by picking ten individual isolated colonies as described in the “Materials and methods” section, and the strains were allowed to accumulate mutations by growing in complete media for 60 passages (Fig. 1b). Based on the nuclear division time in *A. nidulans* and previous calculations [19, 41] we estimated that nuclei underwent 4032 mitotic divisions. This strategy allowed the 10 populations of each strain to accumulate mutations independently from each other along the passages (Fig. 1b). Samples were taken at time 0 (controls) and at the end of the MA experiment for WGS.

**Fig. 2:**
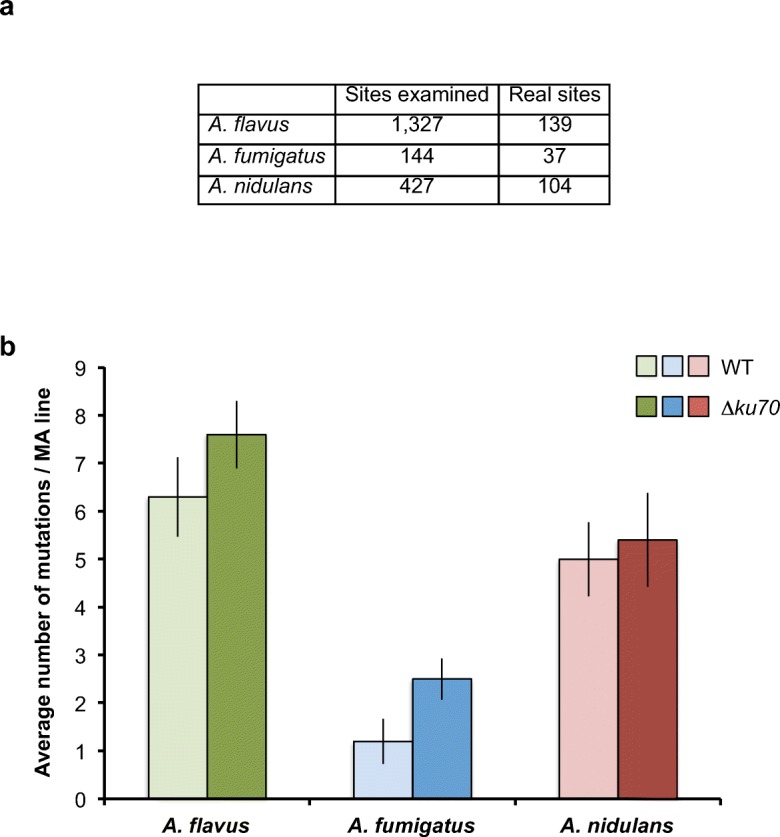
Number of mutations accumulated in the MA lines after 4032 mitosis. **a** Table of the total number of mutations found in the three species before and after filtering. **b** Average number of mutations per MA line in the wild-type and the *∆ku70* mutant strains in all three *Aspergillus* species. Lighter colours represent wild-type strains (WT), and darker colours represent the *∆ku70* mutants of each species. Error bars depict the standard error of the mean

The starting strains before the MA lines were established, together with 2 of the 10 independent MA lines set at the beginning and all the 10 independent MA lines after the 60 growth passages were employed for DNA isolation. There were several reasons for sequencing the populations at passage 0. First, the ∆*ku70* deletion strain of *A. fumigatus* is in the strain D141, a *mat1-1* isolate (rather than in the *mat1-2* strain Af293, which was the sequenced one). Sequencing of the controls at time 0 solved this issue and also provided the sequence of D141 strain, which was largely syntenic to the Af293 genome. In the case of *A. nidulans*, the strains employed in this work are derived from the sequenced wild-type strain FGSC4. The DNA from all the populations was sequenced with 100× coverage on average. This coverage was selected to allow the identification of mutations that were present at low frequency and not fixed in the (nuclear) populations yet. Previous reports using average coverages of 44× and 62×, which are lower than ours, calculated a false-positive error close to zero by Sanger sequencing [42, 43]. Using this strategy, we were able to identify mutations with a frequency as low as 10% that were unique to one population (Fig. 2a).

### Mutation rate

Globally in the MA lines, we identified a total of 122 single nucleotide variants (SNVs) and 17 insertions/deletions (indels) in *A. flavus*, 27 SNVs and 10 indels in *A. fumigatus* and 75 SNVs and 29 indels in *A. nidulans*. Out of those mutations, the wild-type MA lines accumulated 58 SNVs and 5 indel in *A. flavus*, 10 SNVs and 2 indels in *A. fumigatus* and 32 SNVs and 18 indels in *A. nidulans*. *Aspergillus* species like other filamentous fungi are multicellular and multinucleated organisms, i.e. the hyphal filament consists of connected cell compartments, each containing several nuclei (Fig. 1a). Consequently, for the estimation of the mutation rate, we need to consider nuclear divisions (mitoses) rather than cell divisions as previously employed for unicellular eukaryotes [42–47]. As mentioned above, the estimation of the number of mitoses is complicated in the case of filamentous fungi. Our calculations of the number of mitoses are based on previous cell cycle studies performed by Bergen and Morris on liquid media [41], which did not differ substantially from studies by Kaminskyj and Hamer [48] on solid media. The number of nuclear divisions occurring during conidiophore development was not taken into account because the division times and nuclear dynamics in the stalk and vesicle cells are not well characterized. This is partially compensated by the fact that the samples for the cultures passages were taken from the conidiation edge of the colony, which is far behind from the real growing colony front. This strategy to estimate the number of mitoses during the growth passages has been previously carried out by Schoustra et al. [19]. This approach gave a total mutation rate of 4.2 × 10^−11^ ± 5.6 × 10^−12^ (*A. flavus*), 1.1 × 10^−11^ ± 4.1 × 10^−12^ (*A. fumigatus*) and 4.1 × 10^−11^ ± 6.4 × 10^−12^ (*A. nidulans*) per base per mitosis and an average mutation rate for all three species of 3.1 × 10^−11^ ± 5.4 × 10^−12^ per base per mitosis revealing that indeed *Aspergillus* genomes are very robust in comparison to yeasts and higher eukaryotes. The distribution of mutations across the MA lines was consistent with a uniform distribution (Kolmogorov-Smirnov test (K-S test); *P* = 0.93, 0.31 and 0.91 for *A. flavus*, *A. fumigatus* and *A. nidulans*, respectively).

The *∆ku70* deletion mutants of the three species accumulated a slightly higher number of mutations: 64 SNVs and 12 indels in *A. flavus*, 17 SNVs and 8 indels in *A. fumigatus* and 43 SNVs and 11 indels in *A. nidulans* (Fig. 2b), which gives an estimate of a total mutation rate of 5.1 × 10^−11^ ± 4.7 × 10^−12^, 2.2 × 10^−11^ ± 3.8 × 10^−12^ and 4.5 × 10^−11^ ± 8.1 × 10^−12^ per base per mitosis for *A. flavus*, *A. fumigatus* and *A. nidulans*, respectively, and an average mutation rate for all three species of 3.9 × 10^−11^ ± 5.5 × 10^−12^ per base per mitosis. The distribution of mutations across the MA lines of the *∆ku70* mutants was also consistent with a uniform distribution (K-S test; *P* = 1.00, 0.77 and 0.97 for *A. flavus*, *A. fumigatus* and *A. nidulans*, respectively). The differences between the wild-type and the *∆ku70* mutant strains for each species are not statistically significant (two-way ANOVA with Tukey’s post hoc test, *P* = 0.675 for each pair of strains; two-tailed *t* test, *P* = 0.33, *P* = 0.14 and *P* = 0.67 for *A. flavus*, *A. fumigatus* and *A. nidulans*, respectively). However, if we did not consider the different species, and we take into account only the genetic background combining the data from the three wild-type strains and the data from the three *∆ku70* strains, then the differences between the wild-type and the mutant backgrounds are statistically significant (two-way ANOVA with Tukey’s post hoc test, *P* = 0.025), suggesting that the NHEJ has a role in the genome stability of aspergilli.

### Distribution of mutations

The concept of an individual in filamentous fungi is a matter of debate and the definition of a physical individual has been even considered a “hopeless task” [17]. Therefore, we considered our MA lines as populations of nuclei. Thanks to the high coverage of the sequencing, it was possible to detect mutations present in as low as 10% of the nuclei. A boxplot representation of the frequency of all the mutations in the six strains showed that there were a larger proportion of mutations that were not fixed in the *∆ku70* mutants than in the wild-type strain, suggesting that the mutations in the *∆ku70* mutants keep appearing at a slightly faster rate than in the wild-type strain (Additional file 1: Figure S1).

The distribution of single nucleotide mutations in the eight chromosomes appeared to be random and not statistically different from the expected number according to their sizes (K-S test; *P* = 0.92 and 0.92 for *A. fumigatus* wild-type and *∆ku70* mutant, and 0.92 and 1.00 for *A. nidulans* wild-type and *∆ku70* mutant, respectively) (Additional file 2: Figure S2). In the particular case of *A. flavus*, this analysis was complicated because the 138 contigs are not assigned to specific chromosomes. We tried to map these contigs to the chromosomes of the closely related species *A. oryzae* with little success. Therefore, we performed the analysis asking the question whether the mutations were uniformly distributed in the 138 contigs using the K-S test. In this case, we found that *P* < 0.001, meaning that the mutations were not uniformly distributed. Further analysis revealed that all the mutations were located in 20 contigs, which account for 99.25% of the total length of the genome, while the other 118 contigs only provide the remaining 0.75% of the genome, and consequently represent a minor portion of the genome. We repeated the test using only the 20 big contigs accounting for 99.25% of the genome and containing the mutations. In this case, *P* = 0.68 suggests that the distribution of mutations is random in these 20 contigs and in the genome of *A. flavus*.

### Single nucleotide mutations and spectrum

**Fig. 3:**
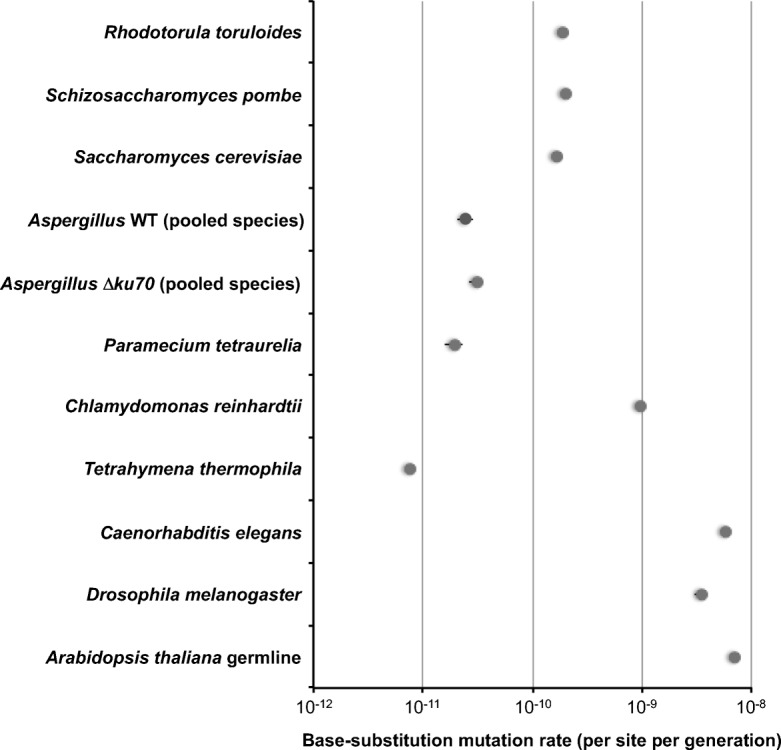
The spontaneous mutation rate in *Aspergillus* species compared to other eukaryotes. The data of *Aspergillus* species is presented as an average of the three species under study for the wild-type and the *∆ku70* mutant strains. The data from the other eukaryotes were obtained from [42–47, 49, 79–83]. Data shown refers to base-substitution mutations

Out of the 280 total number of mutations, we identified a total of 224 substitutions after filtering and confirmation by visual inspection, of which 58, 10 and 32 were found in wild-type *A. flavus*, *A. fumigatus* and *A. nidulans*, respectively, and 64, 17 and 43 were found in the *∆ku70* mutants of *A. flavus*, *A. fumigatus* and *A. nidulans*, respectively. That gives a base-substitution mutation rate of 3.9 × 10^−11^ ± 4.6 × 10^−12^ (*A. flavus* WT), 8.9 × 10^−12^ ± 3.2 × 10^−12^ (*A. fumigatus* WT) and 2.6 × 10^−11^ ± 4.7 × 10^−12^ (*A. nidulans* WT) per base per mitosis, which is one to two orders of magnitude lower than in ascomycete and basidiomycete yeasts [42, 43, 46, 47, 49]. The base-substitution rate for the *∆ku70* mutants was slightly higher: 4.3 × 10^−11^ ± 3.5 × 10^−12^ (*A. flavus ∆ku70*), 1.5 × 10^−11^ ± 3.3 × 10^−12^ (*A. fumigatus ∆ku70*) and 3.5 × 10^−11^ ± 7.9 × 10^−12^ (*A. nidulans ∆ku70*) per base per mitosis, but still lower than in the yeasts (Fig. 3).

**Fig. 4:**
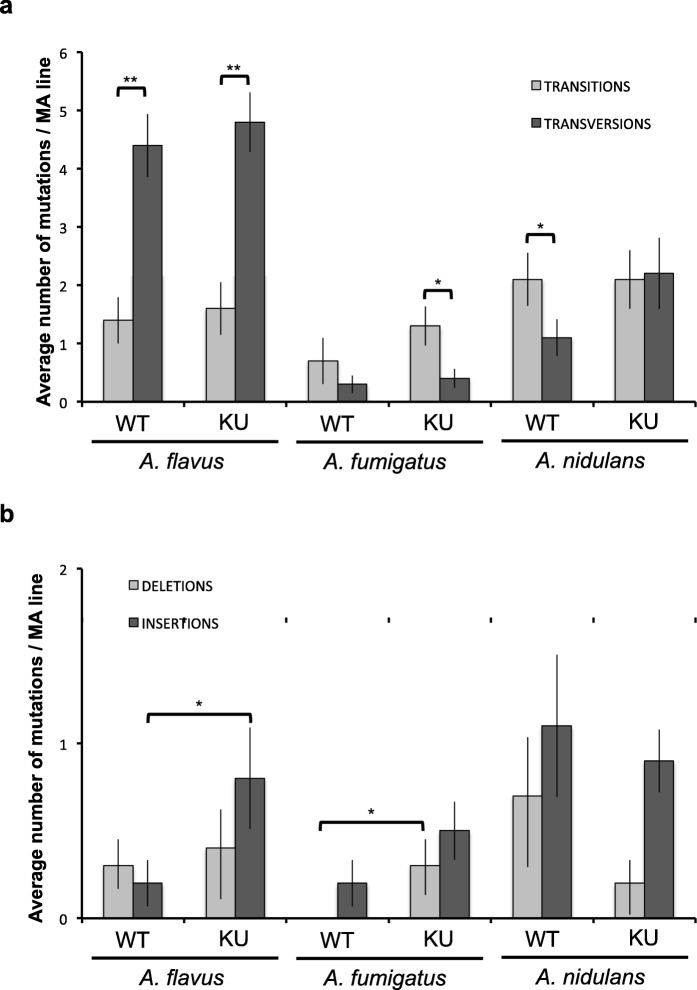
The spectrum of mutation types differs between the three *Aspergillus* species. **a** Average number of base-substitution mutations in the wild-type (WT) and *∆ku70* mutant (KU) of each species. In *A. flavus*, the number of transversions is higher than the number of transitions, opposite to what was found in *A. fumigatus*. In *A. nidulans*, the number of transitions is higher than the number of transversions in the wild-type strain but not in the *∆ku70* mutant. **b** Average number of indel mutations in the wild-type and *∆ku70* mutant of each species. The number of indels is higher in *A. nidulans* than in the other two aspergilli, but in this case, the *∆ku70* mutation seems to protect the genome against indels opposite to what happens in the other two aspergilli. Error bars show the standard error of the mean. Statistically significant differences are shown with *P < 0.05 and **P < 0.01 (one-tailed *t* test)

We found the spectrum of base-substitution mutations varied enormously between the species. The transition/transversion (ts/tv) ratio was extremely low in *A. flavus* (0.3) and higher in *A. fumigatus* (2.3) and *A. nidulans* (1.9). However, the number of base-substitution mutations in *A. fumigatus* was very low. The number of transitions and transversions and the ts/tv ratio did not change significantly between the wild-type and the *∆ku70* mutant strains (Fig. 4a). The major changes found in the *∆ku70* mutants with respect to the wild-type strains were that the difference between the number of transitions and transversions was statistically significant in *A. fumigatus ∆ku70* (two-tailed *t* test; *P* = 0.027) but not in the wild type (two-tailed *t* test; *P* = 0.36). In *A. nidulans*, the number of transversions (22) increased to reach nearly the number of transitions (21) in the *∆ku70*, and consequently, the ts/tv ratio was close to 1.

**Fig. 5:**
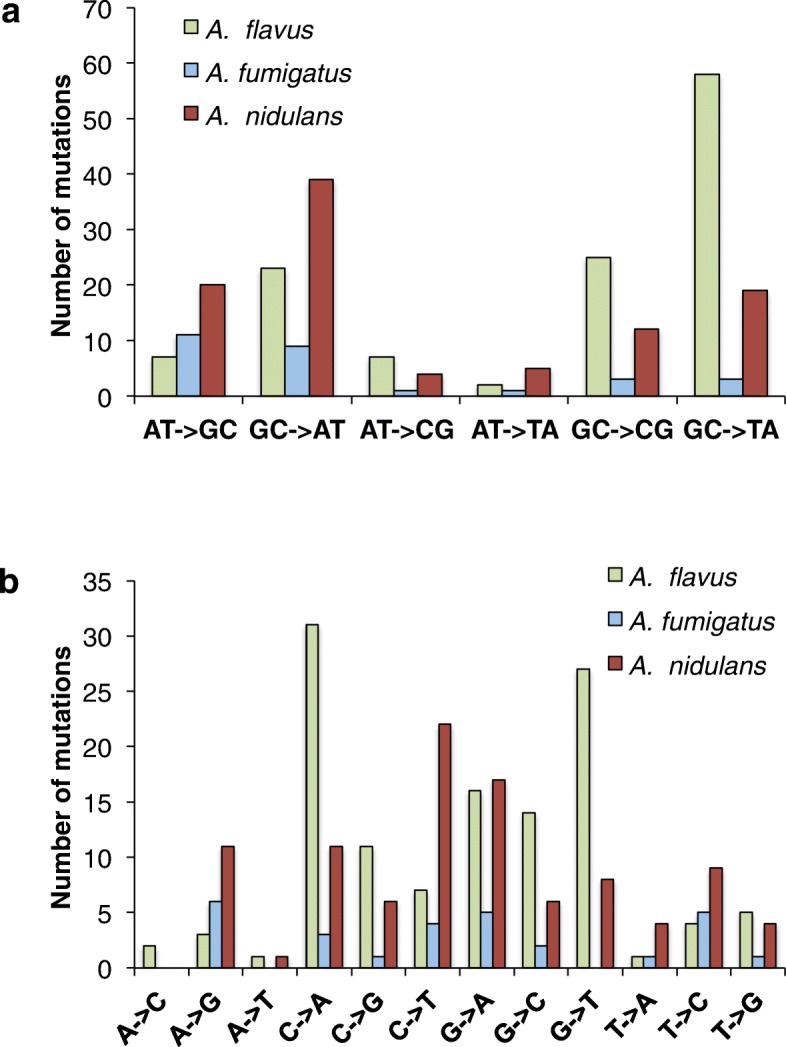
The spectrum of base-substitution mutations differs between the three *Aspergillus* species. **a** The total number of mutations for the six possible transitions and transversions during the evolution of the six strains of *aspergilli*. **b** All possible 12 substitutions in the six strains. Changes of C or G are particularly elevated in *A. flavus* and *A. nidulans*

Considering the very low ts/tv ratio in *A. flavus*, we note that this species produces the toxic and mutagenic secondary metabolite aflatoxin, well known for its induction of G→T and C→A transversions in human p53 gene [50, 51]. Following this fact, it was interesting to observe a strong G/C to A/T bias in *A. flavus* (Fig. 5a). Specifically, there were 5.8 times more G/C to A/T mutations than A/T to G/C mutations. This strong bias was not observed in *A. fumigatus* (1.0×) or *A. nidulans* (2.4×). This mutation bias towards A/T seemed to have been observed previously in many other species ([44]). However, it is only found in *A. flavus* and not in the other two *Aspergillus* species, which do not produce aflatoxin, although *A. nidulans* produces the aflatoxin precursor sterigmatocystin. In *A. flavus* MA lines, the GC→TA transversions are particularly enriched (*χ*^2^; *P* < 0.001; df = 5), while in *A. nidulans* MA lines the GC→AT transitions are the most frequent substitutions (*χ*^2^; *P* < 0.001; df = 5). The most frequent SNPs were 31 times C→A and 27 times G→T in *A. flavus*, 6 times A→G, 5 times G→A and T→C in *A. fumigatus* and 22 times C→T and 17 times G→A in *A. nidulans* (Fig. 5b). According to these estimates, the genomes of *A. flavus* and *A. nidulans* have not reached a nucleotide-content equilibrium. The expected G + C% would be 15% in *A. flavus* and 29% in *A. nidulans* based solely on the base-substitution mutation rate. However, the G + C% content is 50.5% and 52.2% in the genic regions and 45.1% and 47.4% in the intergenic regions of *A. flavus* and *A. nidulans*, respectively. Since the differences between the expected and the observed G + C were very different, we further looked at the frequency of G + C in the third position in the triplet of the fourfold degenerate codons. A plot of the G + C% of each gene vs the G + C% of the synonymous sites revealed that the genome of *A. flavus* shows a higher dispersion of the data with a lower coefficient of determination (Additional file 3: Figure S3). The regression coefficient (slope) in *A. flavus* is also the lowest of the three, suggesting that *A. flavus* has the lowest bias towards G + C of the three species, which is in agreement with the observed data.

**Fig. 6:**
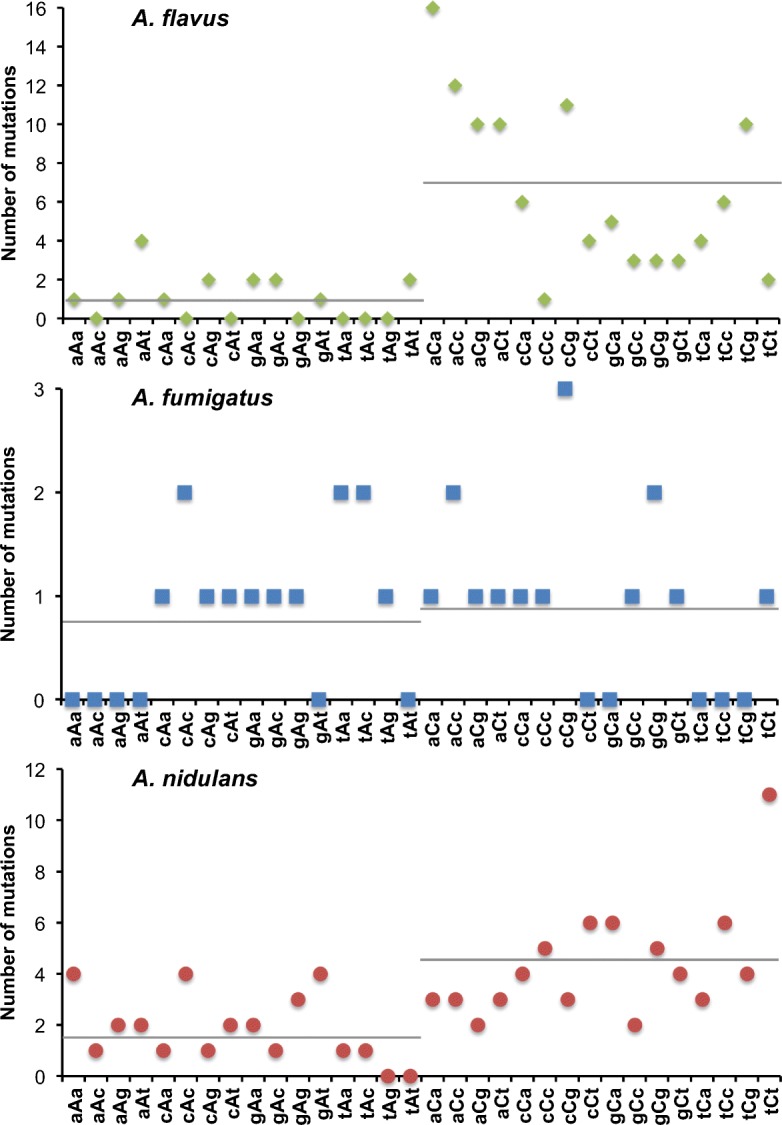
Number of context-dependent mutations in the three *Aspergillus* species. Mutations in CG and CT are particularly enriched in *A. flavus* and *A. nidulans*, respectively. Horizontal bars represent the mean of the number of mutations

The analysis of the trinucleotide context can provide information about the causes of the mutations. Therefore, we also analysed the trinucleotide context of the base-substitution mutations, considering the nucleotides adjacent immediately before and after to the mutated nucleotide. This gives 64 possible combinations that can be reduced to 32 possibilities combining strand orientation nucleotides [46]. The mutated nucleotide together with the neighbouring positions can be assigned to one of these 32 trinucleotides. From this, it was clear that G/C were mutated on average 6.6 and 2.4 times more than A/T in *A. flavus* and *A. nidulans*, respectively (Fig. 6). Although in *A. flavus* the trinucleotide that was most frequently mutated was ACA, the CpG environment accounted for 28% of the base-substitution mutations, while NCA accounted for 25%. In *A. nidulans* however, the most frequent trinucleotide was TCT, and NCT accounted for 24%, which was far higher than the 14% of mutations observed in CpG.

**Fig. 7:**
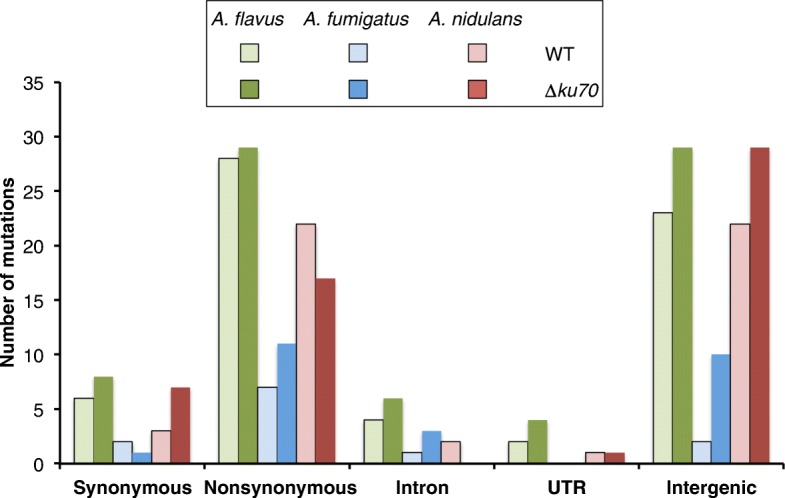
Mutations classified by functional category in the six *Aspergillus* strains

Most of the mutations in all the species were either non-synonymous (missense) or intergenic (Fig. 7, Additional file 4: Figure S4). In Additional file 5: Table S1, Additional file 6: Table S2 and Additional file 7: Table S3, there is a list with the genes found to contain mutations in our MA experiment. When we compared the total number of mutations in the wild-type strains with the *∆ku70* strains for each species, we found only a clear trend in the intergenic mutations (23 in the wild type vs 29 in the *∆ku70*, 2 vs 10 and 22 vs 29 in *A. flavus*, *A. fumigatus* and *A. nidulans*, respectively). The ratio of synonymous to non-synonymous mutations was 0.21, 0.29 and 0.14 in the wild-type strains of *A. flavus*, *A. fumigatus* and *A. nidulans*, respectively, and 0.28, 0.09 and 0.41 in the *∆ku70* mutants of *A. flavus*, *A. fumigatus* and *A. nidulans*, respectively.

### Indel mutations

Out of the 280 total number of mutations, 56 indel mutations were identified in the six strains of *Aspergillus*, 19 were deletions and 37 were insertions, which on average accounts for 1.9 times more insertions than deletions (Fig. 4b). The distribution varied between species, being *A. nidulans* the species accumulating more indel mutations (7 deletions and 11 insertions in the wild-type strain). This is 1.8 times lower than the number of base-substitution mutations. The low proportion of indel mutations found in aspergilli (ranging from 0.08 to 0.36) is consistent with other organisms. In general, indels were observed to be increased in *∆ku70* mutants compared to the corresponding wild-type strain. However, insertions in the *∆ku70* were only significantly increased in *A. flavus* (one-tailed *t* test; *P* = 0.038) and deletions in *A. fumigatus* (one-tailed *t* test; *P* = 0.033). In *A. nidulans*, the number of indel mutations was even higher in the wild type (18 indel mutations) than in the *∆ku70* mutant (11 indel mutations), with a particular but not significant increase in the number of deletions (7 in the wild type vs 2 in the *∆ku70* mutant strain) (one-tailed *t* test; *P* = 0.09). Based on these data, we estimated an indel mutation rate of 3.4 × 10^−12^ ± 1.5 × 10^−13^ (*A. flavus*), 1.8 × 10^−12^ ± 1.2 × 10^−13^ (*A. fumigatus*) and 1.5 × 10^−11^ ± 4.4 × 10^−13^ (*A. nidulans*) per base per mitosis for the wild-type strains and 8.1 × 10^−12^ ± 2.2 × 10^−13^ (*A. flavus*), 7.1 × 10^−12^ ± 2.2 × 10^−13^ (*A. fumigatus*) and 9.1 × 10^−12^ ± 1.5 × 10^−13^ (*A. nidulans*) per base per mitosis for the *∆ku70* mutant strains.

### Cessation of cleistothecia development in the sexual MA lines of the *A. nidulans* NHEJ mutants

**Fig. 8:**
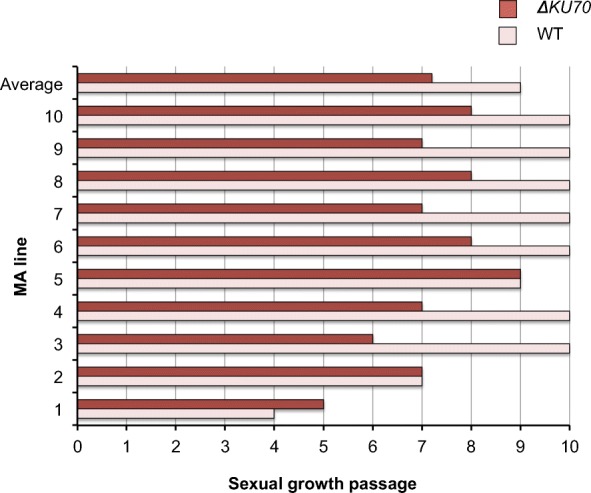
Formation of cleistothecia was halted in the MA lines during the sexual passages of *A. nidulans*. When the MA lines were going through homotallic meiotic passages, the formation of the sexual structures cleistothecia started to halted after 4 passages. None of the *∆ku70* MA lines reached 10 sexual passages

*A. nidulans* is the only homothallic species of these three. Self-crossing provides a framework in which any mutations or changes in genome sequence or structure that arise during the meiotic divisions are not due to the genetic heterogeneity of two parental strains. Taking advantage of this characteristic, a similar procedure was carried out with *A. nidulans* during sexual reproduction. We employed the same 10 independent MA lines established for the asexual passages. This scenario combined mitotic with meiotic divisions. As it can be observed in Fig. 8, all the *∆ku70* MA lines stopped producing cleistothecia after 5–9 passages. On the other hand, most of the wild-type MA lines (7 out of 10) still produced cleistothecia after 10 passages. We can assume that the cessation of cleistothecia production was not a consequence of the accumulation of mutations, as only 15 mutations were identified in 9 populations of the *∆ku70* strain after 5 passages and 5 mutations were found in the wild-type MA lines, and none of them seem to play an essential role (Additional file 8: Table S4). Therefore, the causes of cessation in the production of cleistothecia could be either epigenetics, or big chromosomal re-arrangements, or shortening of the telomeres. Although null mutations in histone modification genes associated with defects in reproduction also show vegetative growth defects and mutants in the DNA methyltransferase still produce cleistothecia [52], hypomorphic mutations could be responsible for this inhibition (see the “Discussion” section for other explanations). Although it is easy to argue how either chromosomal re-arrangements or telomere shortening could result in sterile cleistothecia (empty cleistothecia, without ascospores), it is not obvious how they could inhibit cleistothecial formation.

**Fig. 9:**
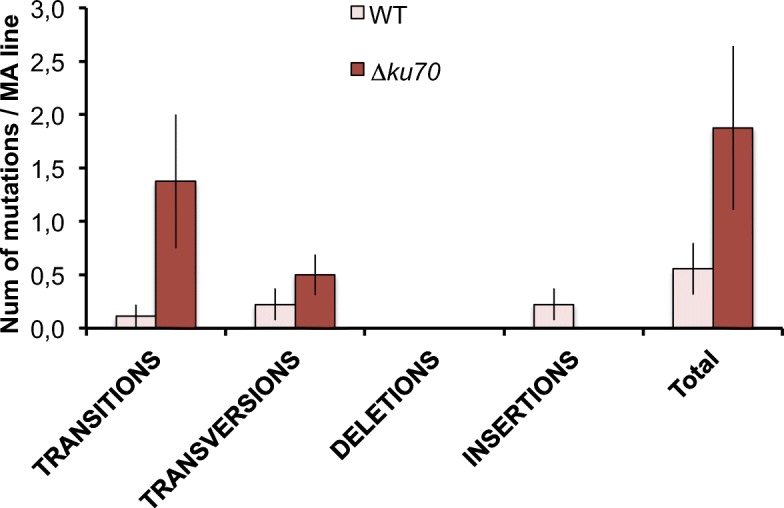
Number and type of mutations in the sexual MA lines. The number of mutations was slightly higher in the *∆ku70* strain, although it was not statistically significant (one-tailed *t* test, *P* = 0.052). This increase is mainly due to elevated number of transitions (one-tailed *t* test, *P* = 0.026)

After the 5 meiotic rounds and the subsequent WGS, we found a total of 20 mutations, 5 in the wild-type strain and 15 in the *∆ku70* mutant strain (Fig. 9). Even though the total number of mutations is higher in the *∆ku70*, this is not statistically significant (one-tailed *t* test; *P* = 0.052). We identified a total of 2 indels, which were all accumulated in the wild-type strain. The number of base-substitution mutations was significantly higher in the *∆ku70* mutant strain (one-tailed *t* test; *P* = 0.028) with 15 mutations vs 3 mutations in the wild-type strain. The differences are mainly due to an increase number in transitions (1 in the wild-type vs 11 in the *∆ku70* strain). However, only one of the MA lines is the major contributor with 5 base-substitution mutations. This is the MA line number 5, which was capable of reaching the maximum number of sexual passages of all MA lines. Despite that the MA line 5 accumulated a higher number of mutations, the distribution of mutations across the MA lines was consistent with a uniform distribution (K-S test), *P* = 0.08 and 0.93 for the wild-type and the *∆ku70* strains, respectively.

## Discussion

Filamentous fungal species are excellent cell factories for the production of biotech goods such as organic acids, recombinant enzymes and pharmaceuticals. This makes them worth billions of euros every year. This is the first report that estimates the mutation rate based on WGS in filamentous fungi. We allowed 10 independent MA lines of each strain to accumulate mutations for over ~ 4000 mitoses with 60 growth passages. Under these conditions, we assumed that the nuclei with deleterious mutations were lost during the experiment, and only nuclei with mutations that can grow in complete media will progress. For this reason, we chose complete media rather than minimal media, which would have resulted in a lower accumulation of mutations due to the loss of many auxotrophic mutants. Globally, our experiment considered a total of ~ 242,000 mitoses and we sequenced the whole genomes with a 100× depth on average. After the analysis of ~ 1.9 × 10^11^-bp sequences, we have estimated that the total mutation rate is 3.1 × 10^−11^ ± 5.4 × 10^−12^ per base per mitosis on average for the three *Aspergillus* strains with small differences between the different species (4.2 × 10^−11^ ± 5.6 × 10^−12^ per base per mitosis in *A. flavus*, 1.1 × 10^−11^ ± 4.1 × 10^−12^ per base per mitosis in *A. fumigatus* and 4.1 × 10^−11^ ± 6.4 × 10^−12^ per base per mitosis in *A. nidulans*). Only the mutation rate of *A. fumigatus* seems to be statistically different from the other two *Aspergillus* species (two-way ANOVA, Tukey’s post hoc test *P* = 2.9 × 10^−5^ and 2.5 10^−3^ with *A. flavus* and *A. nidulans*, respectively). We considered two possible scenarios for these differences. First, the genome of *A. fumigatus* is more robust. However, it is hard to argue that this is due to its pathogenic capabilities, as it is an opportunistic pathogen and *A. flavus* is also a pathogenic species. The second and more likely possibility is that many calls in regions of poor/spurious read alignments in *A. fumigatus* were filtered out, resulting in a lower number. This second possibility is supported by the coverage depth of 97% in *A. nidulans* and only 90% in *A. fumigatus*.

The rate of non-homologous integrations of recombinant constructs during transformation is usually very high in filamentous fungi, which can make the construction of knock-out mutants, for example, a very laborious work. For this reason, most laboratories across the world have established the use of NHEJ mutants to increase the rate of the homologous integration [25, 28, 32, 53–61]. However, the genomic stability of these strains has not been tested up to date and raised concerns of the appropriateness of using these mutants in industry or to assess virulence in pathogenic fungi. Here, we found that the *∆ku70* deletion mutants accumulated an average of 1.2 times higher number of mutations: 76, 25 and 54 in all the MA lines. We estimated a total mutation rate of 5.1 × 10^−11^ ± 4.7 × 10^−12^ (*A. flavus*), 2.2 × 10^−11^ ± 3.8 × 10^−12^ (*A. fumigatus*) and 4.5 × 10^−11^ ± 8.1 × 10^−12^ (*A. nidulans*) per base per mitosis. The average mutation rate for all three species is 3.9 × 10^−11^ ± 5.5 × 10^−12^ per base per mitosis. This number, although statistically significant, did not increase drastically for mutations accumulated in *∆ku70* strains compared to the wild-type strains. Bruggeman et al. estimated a base-substitution mutation rate of 2.26 × 10^−10^ per base per mitosis for asexual passages of *A. nidulans* based solely on fitness assays [21]. Our estimate using WGS is 10× lower (2.5 × 10^−11^ ± 4.2 × 10^−12^ per base per mitosis). When we compared the *Aspergillus* average base-substitution mutation rate with unicellular fungi (Fig. 3), we found that it is one order of magnitude lower than the reported mutation rates for yeasts: 1.70 ± 0.13 × 10^−10^ or 2.00 ± 0.10 × 10^−10^ per base per generation in *Schizosaccharomyces pombe* [42, 43], 1.67 × 10^−10^ per base per generation in *Saccharomyces cerevisiae* [46] and 1.90 × 10^−10^ per base per generation in the basidiomycete yeast *Rhodotorula toruloides* [47]. Even more important, the base-substitution mutation rate in the *∆ku70* mutants is still 5.3–6.4 times lower than in the yeasts. *S. cerevisiae* is frequently employed as cell factory and also is very important in fermentation processes to make bread, beer or wine. Another cell factory bacterium *Escherichia coli* has a base-substitution mutation rate of 2.6 × 10^−10^ per base per generation based on reporter constructs [62], the same range as yeasts but an order of magnitude higher than the wild-type *Aspergillus* and 8.3 times higher than the *∆ku70* mutants. Taking altogether, this suggests that *Aspergillus* genomes are the most robust genomes of the cell factories analysed so far and that the *∆ku70* mutants have genomes that are robust enough for use directly in industrial fermentation processes.

The fact that the number of mutations is slightly higher in the *∆ku70* than in the wild-type strains raises some questions about alternative roles of the KU70/KU80 dimer in genome stability. After a DNA double-strand break, two possible repair mechanisms can act: the NHEJ and the HR [63, 64]. Many filamentous fungi are haploids during most of their life cycles, although diploids can be constructed and maintained in the lab. Therefore, in the absence of a sister chromatid and a homologous chromosome, the NHEJ is responsible for the repair of DSBs during the G1 and early S phases [63, 64]. However, in the absence of KU, cells are still capable of DNA resection in G1 at DSBs prior to repair by homologous recombination, which activates the checkpoints. Bergen and Morris [41] estimated that the G1 phase lasts 15 out of 75 min of the complete nuclear cycle of *A. nidulans*. Therefore, *Aspergillus* species spend most of their time in S or G2 phases (55 min) where sister chromatids are present and HR is possible. Indeed, the number of base-substitution mutations was on average 24% higher in the *∆ku70* than in the wild-type strain (ranging from 10 to 70%). This is in agreement with the time that *Aspergillus* spends in G1, which is approximately 20% of the total nuclear cycle. Furthermore, it has been shown in yeasts that the replication fork is stalled at fragile sites containing CCG and CAG trinucleotides that lead to DSBs [65]. However, when we analysed the trinucleotide context of the mutations found in our MA lines, we found that only in the case of *A. flavus* mutations in the CCG trinucleotides are statistically significant (*χ*^2^; *P* = 0.001; df = 1). Actually in *A. flavus* MA lines, mutations in the CpG context are particularly enriched (*χ*^2^; *P* = 0.00; df = 1). In most of the reports describing the spontaneous mutation rates in eukaryotic organisms, mutations in this context are associated mainly to the oxidation of guanine to give 8-oxo-guanidine, which produces a GC→AT transition. These mutations are significantly increased in *A. fumigatus* and *A. nidulans* accounting for 32% and 39% of all the base-substitution mutations (*χ*^2^; *P* = 0.028 and 0.000; df = 1, respectively), but not in *A. flavus* (*χ*^2^; *P* = 0.517; df = 5). This can be easily explained because the strains were cultivated on the surface of solid media and continuously exposed to an air interphase for 8–9 months. Methylation of cytosines results in the spontaneous deamination of cytosines provoking a GC→AT transition. However, the oxidation of guanine is probably more parsimonious taking into account that 5-methylated cytosines (5-mC) have not been detected in neither *A. nidulans* nor *A. flavus* yet [52, 66–68], despite the existence of DNA methyltransferases in the aspergilli genomes.

The type of mutation that most called our attention was the high number of GC→TA transversions found in *A. flavus* MA lines, which accounts for nearly half of all the base-substitution mutations (*χ*^2^; *P* = 0.000; df = 1). As mentioned earlier, *A. flavus* produces the carcinogenic secondary metabolite aflatoxin, the most potent natural environmental mutagen known. Aflatoxin is infamous for causing human hepatocarcinomas by inducing mutations in the p53 oncogene [50, 51]. Specifically, an epoxide derivative of aflatoxin intercalates into DNA and the epoxide moiety reacts with the guanine residue to produce GC→TA transversions [69]. *A. fumigatus*, which is unable to produce aflatoxin, accumulated only 11% of GC→TA transversions. On the other hand, *A. nidulans* produces an intermediate in the aflatoxin pathway, sterigmatocystin, which is also a carcinogen and shows demonstrable mutagenic properties in vitro [70]. Yet evidence does not support a mutational role for sterigmatocystin in nature, likely due to the non-pathogenic nature of *A. nidulans* as well as the low levels of sterigmatocystin synthesized by this species in comparison to aflatoxin output in many *A. flavus* strains (including the strain used in this study which produces aflatoxin in the medium used to grow the strains, [71]). Indeed, in *A. nidulans*, the number of GC→TA transversions accumulated is not significantly increased (*χ*^2^; *P* = 0.500; df = 1). Considering the *A. flavus* data, this raises interesting questions about the consequences and advantages of producing mutagenic secondary metabolites for endogenous producers.

During this study, it was shown that the absence of KU70 lead to an early cessation of cleistothecia development after repetitive selfing crosses in *A. nidulans*. This observation was not reported in a previous article that employed 40 generations of MA lines [21]. However, their setup was slightly different from our setup and this observation could have been overlooked. They observed a reduction in fitness after the 40 generations, which they argued was due to the accumulation of mutations [21]. Another study in which Xu employed the heterothallic mushroom *Agaricus bisporus* [18] reported an inbreeding depression, as observed with the percentage of heterokaryons forming primordia, density of fruiting bodies and other parameters. Because *A. bisporus* is heterothallic, the inbreeding coefficient was 0.5 and yet they observed a significant decreased in the percentage of heterokaryons forming primordia [18]. In our setup, because *A. nidulans* is homothallic, the inbreeding coefficient was 1.0. We observed that 30% of the wild-type MA lines ceased to form cleistothecia suggesting an inbreeding depression. In case of the *∆ku70* MA lines, all ceased to form cleistothecia before the 10 passages. The reason is unclear. Both strains are *veA1*, which made us to consider that this allele could not be responsible for the phenomenon. However, we cannot exclude possible genetic interactions between *veA1* and *∆ku70* leading to the cessation of cleistothecia formation. Theoretically, chromosomal re-arrangements are more likely to be the cause than telomere shortening, because it appears to be specific to meiosis, and therefore the more drastic depression could be due to increased genomic instability in the absence of KU heterodimer in the *∆ku70* strain during meiosis. However, this does not explain why cleistothecia development was halted. The phenomenon resembles epigenetic regulation. Several studies have reported aberrancies in sexual development in *A. nidulans* (or *A. flavus*) chromatin modifying mutants including DmtA (a cytosine methyltransferase, [52]), SntB (a histone reader, [72]), Set3 (a chromatin-associated PHD domain protein, [73]) and HosA (a histone deacetylase, [74]).

## Conclusions

Aspergilli are widely used in the biotechnology industry and are emerging as the best model systems for fungal genetics and comparative genomics with species that are pathogens of plants and/or animals. The increasing capacity of DNA sequencing has allowed us to evaluate the consequences of evolution in a control experiment in the laboratory for the first time in a multicellular eukaryote under standard non-selective conditions. The widespread use of the NHEJ strains has impelled this work. We have found that the genomes of the *Aspergillus ∆ku70* strains are very robust, more than other cell factories employed in the biotech industry, and therefore they can be considered safe enough for production purposes. A stable genome is especially important for biotechnology applications, where much time and money is spent to optimize processes and a small change in yield can have large financial consequences. Therefore, as shown in this work, *Aspergillus* and their corresponding NHEJ mutants also offer robustness in addition to the other previously known advantages for industrial applications. The comparative studies on *Aspergillus* genome evolution and stability observed in this work can be extrapolated to other Aspergilli, and maybe also applicable to other fungi, where the use of NHEJ mutants is growing.

## Materials and methods

### Strains and media used in this study

There are several genes involved in the NHEJ DNA repair mechanism; for this work, only mutants in the homologues of human *KU70* were employed to allow for comparisons between species. All the strains used in this study are listed in Additional file 9: Table S5. Strains were grown in *Aspergillus* complete media [75].

### Establishment of MA lines and growth passages

The MA experiment was performed as previously described [19, 76] with small modifications as depicted in Fig. 1b. Appropriate dilutions of one single stock of spores of each strain were plated on 10 plates per strain containing complete solid media to give single isolated colonies. Ten independent colonies of each strain were point inoculated in the centre of individual plates (one plate per colony). Then, the 10 independent MA lines of each strain were established. Plates were incubated for 3 days at 37 °C (30 °C for *A. flavus*), and the spores were harvested and were kept frozen as controls.

Plugs were taken from the conidiating edge of each single colony, i.e. from the farthest point from the colony centre that was conidiating, just before there was only vegetative mycelium. Plugs were placed in a microcentrifuge tube with 1000 μl of tween 0.05% (0.02% for *A. fumigatus*) to resuspend the spores. For the next growth passage (generation), 2 μl of the suspension was inoculated in the centre of the plate and the plates were incubated at 37 °C (30 °C for *A. flavus*). Two passages were made per week, which accounted for bottlenecks every ~ 58 and ~ 77 nuclear mitosis (3 and 4 days, respectively).

In the case of *A. nidulans*, sexual passages were also performed. The procedure was the same, with the difference that one cleistothecium from each plate was picked, cleaned of spores and Hülle cells, and ascospores were resuspended in 200 μl of tween 0.05% and 0.02% respectively.

### Molecular techniques

After all passaging was completed, gDNA of populations at passage 0 and after 60 passages was isolated using standard techniques. For standard fragment Illumina libraries, 500 ng–1 μg of genomic DNA was sheared using the Covaris E210 (Covaris) and sized selected for 475 bp using Agencourt Ampure Beads (Beckman Coulter). The DNA fragments were treated with end repair, A-tailing and adapter ligation using the TruSeq DNA Sample Prep Kit (Illumina) and purified using Agencourt Ampure Beads (Beckman Coulter). These were sequenced on HiSeq to produce 2 × 150 bp reads. These reads were aligned to the reference genome using BWA [77], and on average, 100× coverage was sequenced for each population. SNPs and small indels were called using bbtools callVariants.sh (https://sourceforge.net/projects/bbmap/) and allele frequencies estimated. The SRA accession numbers for the genome sequences are listed in Additional file 10: Table S6.

### Filtering of mutations

To remove false-positive calls and identify the point mutations that appeared during the growth passages, we removed all calls that had less than 3 supporting reads. We found that in some areas, the sequenced strain was highly divergent from the reference genome deposited in the database. In these cases, only a small percentage of the reads from these regions were able to align, and none of the calls in these regions were reliable. To filter out these unreliable calls, they were also removed if they occurred in regions with less than 10% of the average depth to avoid false positives in highly diverged regions. Finally, since point mutations will be unique to only a single strain, all calls that were shared by multiple strains were removed.

Putative single nucleotide variants and small indels were identified using callVariants (from the bbmap package v37.06) with default parameters and adding “rarity = 0.05 minallelefraction = 0.05”. A series of filters were then applied, only calls unique to a single strain were retained, calls with less than 10% of the average depth were discarded after noting an excess of these calls in regions of poor/spurious read alignment especially in *A. fumigatus* and calls with less than 3 reads supporting the alternate allele were also discarded. The remaining calls were then manually reviewed using the Integrative Genomics Viewer (IGV) [78] genome browser to remove calls that were obviously bad, typically due to sequencing errors. Allele frequency counts were also manually adjusted during the review due to some issues with allele frequency estimates in that version of callVariants. No variants of higher complexity than SNVs and small indels were attempted.

### Statistical analysis

The statistical tests described in the text for the analysis of the data were performed using Excel, PAST or STATGRAPHICS software.

## Supplementary information

**Additional file 1: Figure S1.** Boxplot showing the frequency of each mutation identified in all the MA lines of each strain. MA lines consist of populations of nuclei. The boxplot represents the frequency of each mutation found in each population of nuclei in each MA line. Mutations were called when they were present in more than 10% of nuclei.

**Additional file 2: Figure S2.** The distribution of mutations per chromosome in the *Aspergillus* species. Total number of mutations per chromosome in all MA lines of both wild type and *∆ku70* mutant strains are shown for each species. Black bars represent the chromosomes in which the *ku70* and *ku80* genes are located in each species.

**Additional file 3: Figure S3.** The plot depict the GC (G + C %) in the y-axis vs the GC4fold (G + C% in the third position of the four fold degenerate triplets) for each coding region of the genes in the three species. Formulas represent the linear fit of the data and the coefficient of determination of the fit.

**Additional file 4: Figure S4.** Mutations classified by functional category in the six *Aspergillus* strains.

**Additional file 5: Table S1.** Genes mutated during the MA experiment in *A. flavus.*

**Additional file 6: Table S2.** Genes mutated during the MA experiment in *A. fumigatus.*

**Additional file 7: Table S3.** Genes mutated during the asexual MA experiment in *A. nidulans.*

**Additional file 8: Table S4** Genes mutated during the sexual MA experiment in *A. nidulans.*

**Additional file 9: Table S5.** Strains used in this study.

**Additional file 10: Table S6.** SRA accessions.

## Data Availability

Strains used in this study are listed in Additional file 9: Table S5 and available at the Fungal Genetics Stock Center (http://www.fgsc.net/asperg.html). The last passage of the mutation accumulating lines is available on request to the laboratories (*A. flavus* from Keller lab, *A. fumigatus* from Braus lab and *A. nidulans* from Cánovas lab). The SRA accession numbers for the genome sequences are listed in Additional file 10: Table S6).
