# Supplementary figures and images for: Genome sequencing of evolved aspergilli populations reveals robust genomes, transversions in A. flavus, and sexual aberrancy in non-homologous end-joining mutants
Source: BMC Biol. 2019 Nov 11;17:88. doi: 10.1186/s12915-019-0702-0 (PMC6844060; doi:10.1186/s12915-019-0702-0)

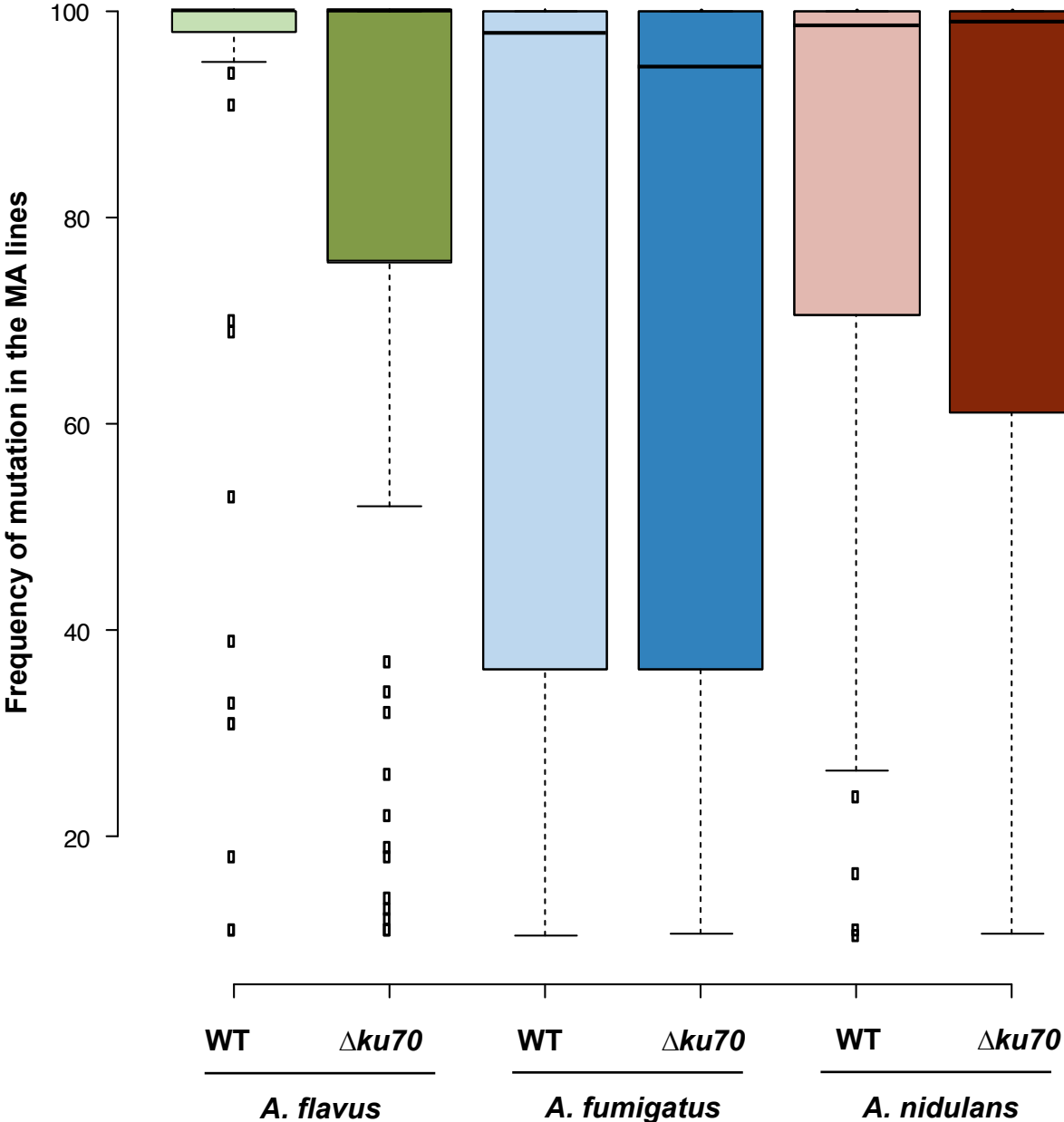

Supplement: Supplementary file 1 — Additional file 1: Figure S1. Boxplot showing the frequency of each mutation identified in all the MA lines of each strain. MA lines consist of populations of nuclei. The boxplot represents the frequency of each mutation found in each population of nuclei in each MA line. Mutations were called when they were present in more than 10% of nuclei. [file 12915_2019_702_MOESM1_ESM.pdf]

**a** *A. fumigatus*

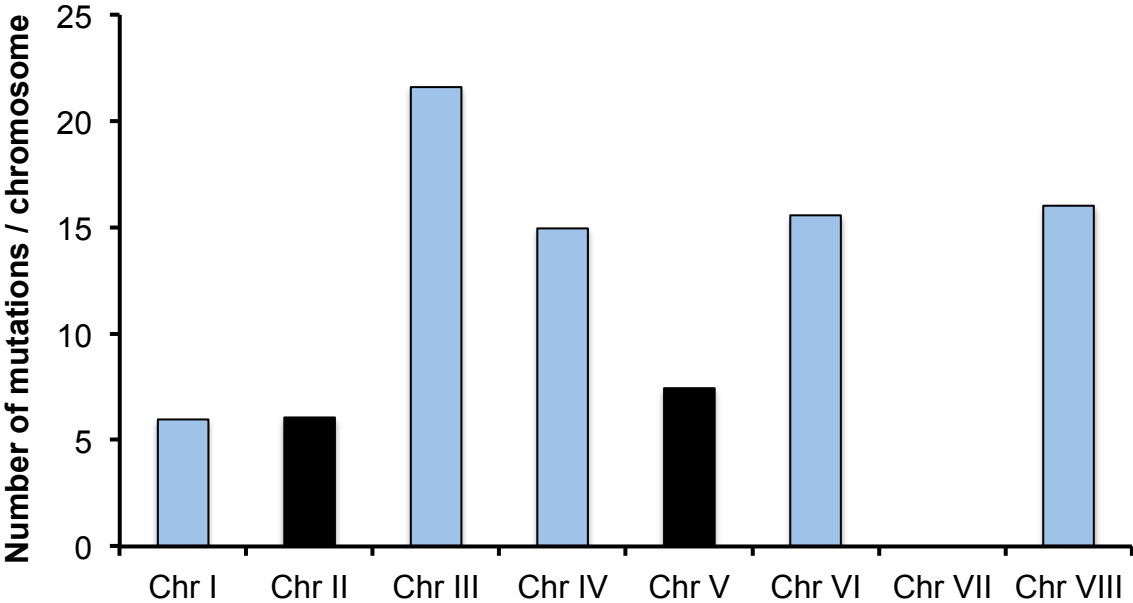

**b** *A. nidulans*

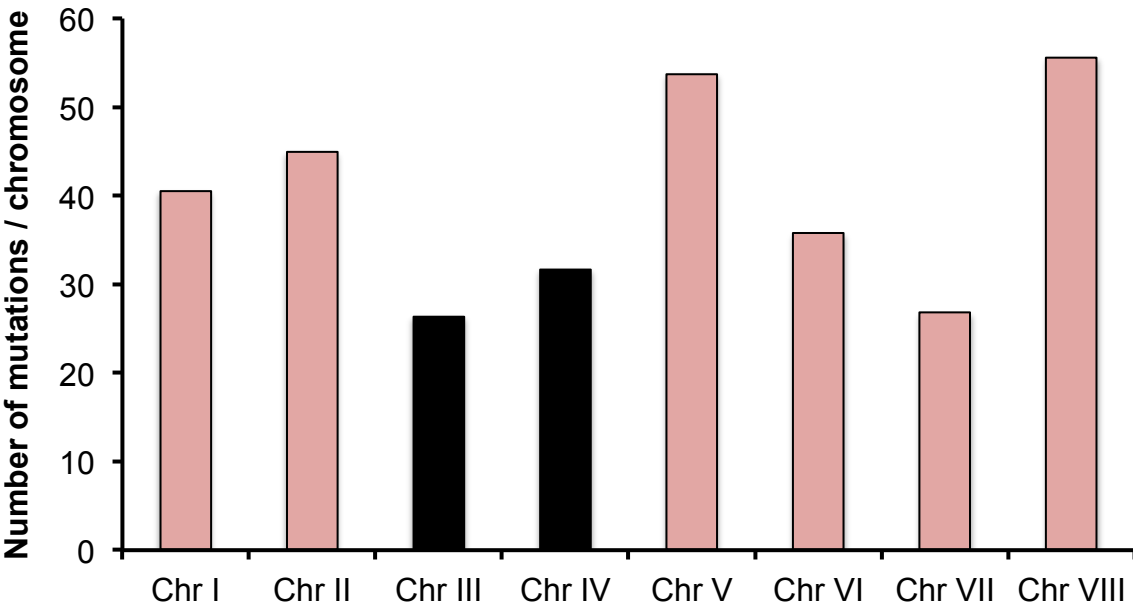

Supplement: Supplementary file 2 — Additional file 2: Figure S2. The distribution of mutations per chromosome in the Aspergillus species. Total number of mutations per chromosome in all MA lines of both wild type and ∆ku70 mutant strains are shown for each species. Black bars represent the chromosomes in which the ku70 and ku80 genes are located in each species. [file 12915_2019_702_MOESM2_ESM.pdf]

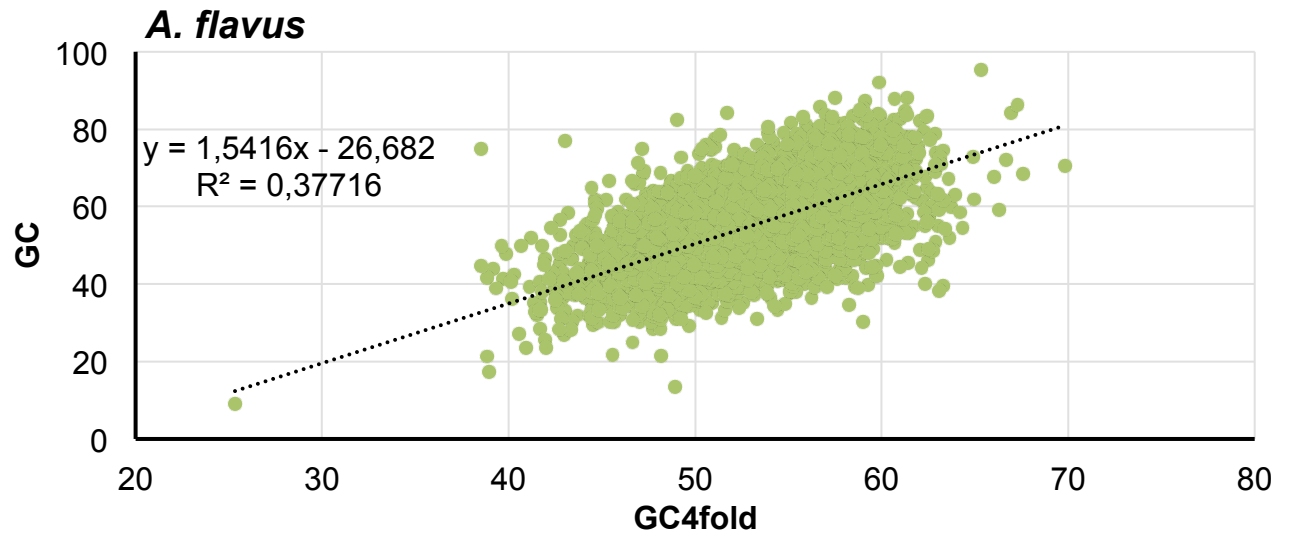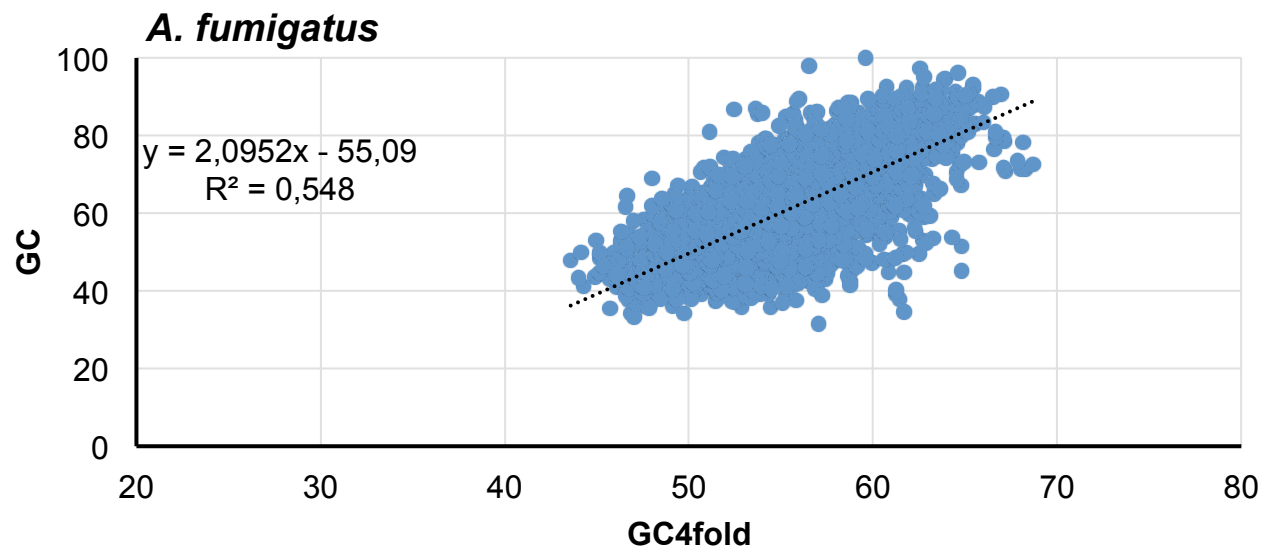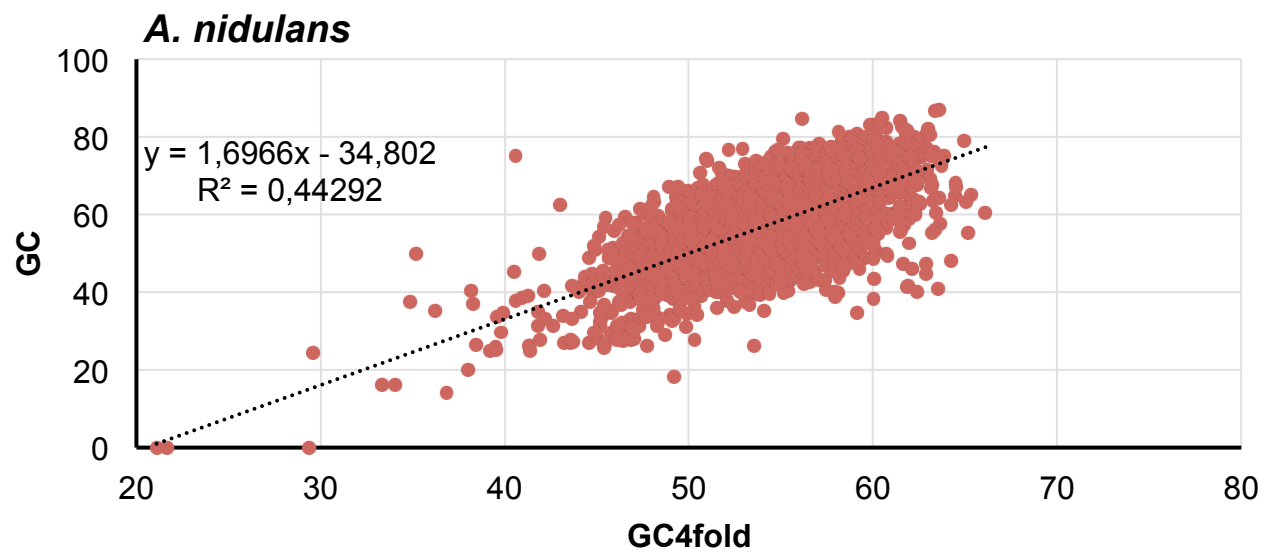

Supplement: Supplementary file 3 — Additional file 3: Figure S3. The plot depict the GC (G + C %) in the y-axis vs the GC4fold (G + C% in the third position of the four fold degenerate triplets) for each coding region of the genes in the three species. Formulas represent the linear fit of the data and the coefficient of determination of the fit. [file 12915_2019_702_MOESM3_ESM.pdf]

Supplementary Figure 4

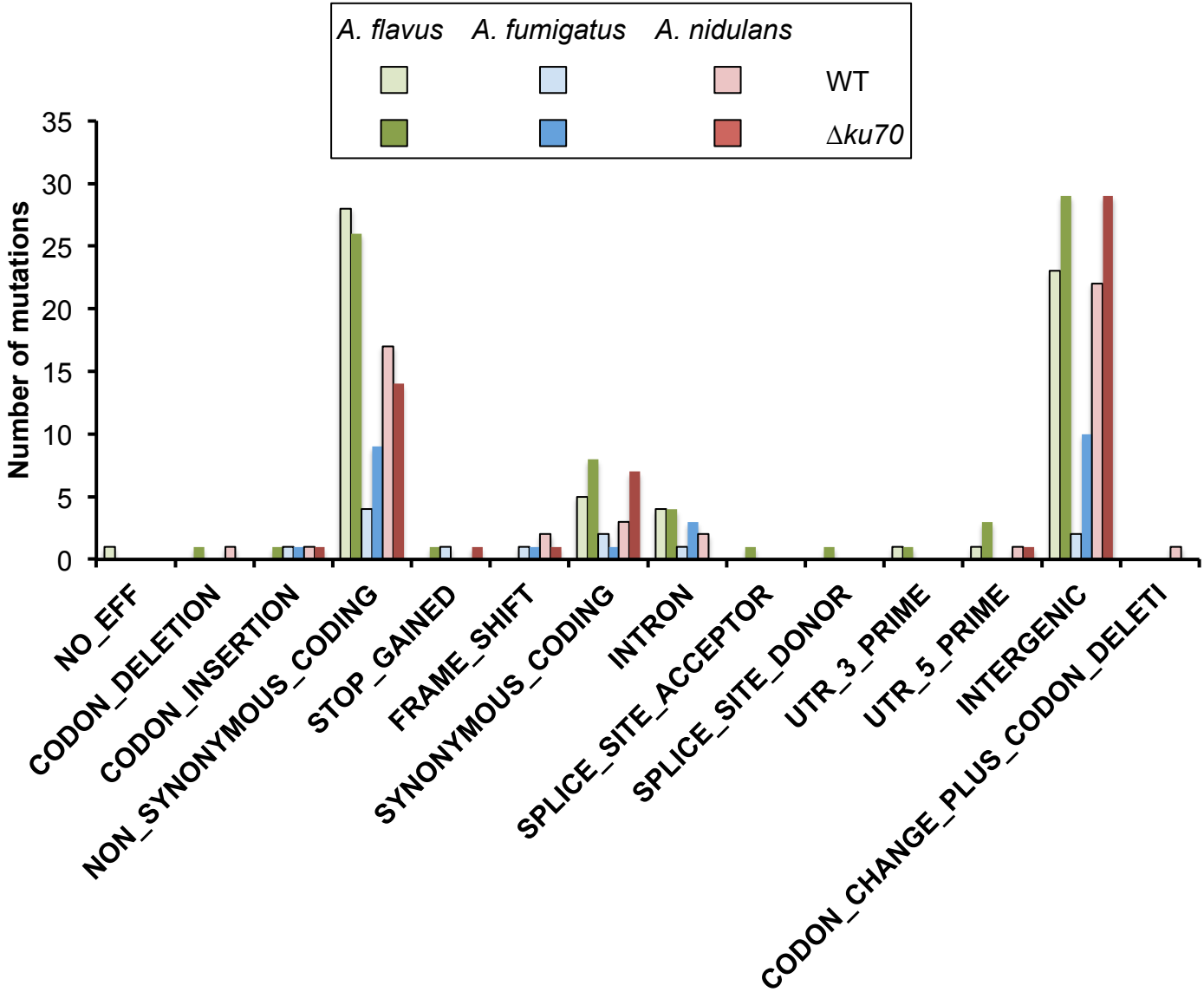

Supplement: Supplementary file 4 — Additional file 4: Figure S4. Mutations classified by functional category in the six Aspergillus strains. [file 12915_2019_702_MOESM4_ESM.pdf]
