# Supplementary material for: Genome sequencing of evolved aspergilli populations reveals robust genomes, transversions in A. flavus, and sexual aberrancy in non-homologous end-joining mutants
Source: BMC Biol. 2019 Nov 11;17:88. doi: 10.1186/s12915-019-0702-0 (PMC6844060; doi:10.1186/s12915-019-0702-0)
Supplement: Supplementary file 5 — Additional file 5: Table S1. Genes mutated during the MA experiment in A. flavus. [file 12915_2019_702_MOESM5_ESM.pdf]

STRAIN AND CONTIG	POSITION	MA LINE	PROTEIN ID	REF	ALT	GENE	ANOTATION
***A. flavus* WT**							
A_flavus_1041045517025	1822808	WT60-1	34471	T	C	AFL2T_00651	Protein of unknown function
A_flavus_1041045517025	2777779	WT60-1	INTERGENIC	G	C		
A_flavus_1041045517025	3622468	WT60-1	35071	C	A	AFL2T_01251	Has domain(s) with predicted role in transmembrane transport and integral to membrane localization
A_flavus_1041045517009	824036	WT60-1	INTERGENIC	AT	A		
A_flavus_1041045517023	1620188	WT60-1	33010	G	C	AFL2T_04441	Protein of unknown function
A_flavus_1041045517014	1422303	WT60-1	INTERGENIC	G	A		
A_flavus_1041045517021	1925828	WT60-1	INTERGENIC	C	A		
A_flavus_1041045517027	424961	WT60-1	36148	G	A	AFL2T_06616	Protein of unknown function
A_flavus_1041045517026	1342483	WT60-1	35806	G	T	AFL2T_09826	
A_flavus_1041045517028	58445	WT60-1	INTERGENIC	G	A		
A_flavus_1041045517011	1706543	WT60-1	INTERGENIC	A	AAC		
A_flavus_1041045517022	336328	WT60-1	31925	G	T	AFL2T_11385	
A_flavus_1041045517024	87669	WT60-1	INTERGENIC	C	A		
A_flavus_1041045517012	234199	WT60-2	INTERGENIC	C	A		
A_flavus_1041045517009	300977	WT60-2	26539	G	C	AFL2T_03043	
A_flavus_1041045517027	94538	WT60-2	36027	C	A	AFL2T_06495	
A_flavus_1041045517027	2214797	WT60-2	36995	G	T	AFL2T_10230	Ortholog(s) have role in cellular iron ion homeostasis and fungal-type vacuole membrane localization
A_flavus_1041045517026	586826	WT60-2	35565	G	T	AFL2T_09585	
A_flavus_1041045517012	3650023	WT60-3	INTERGENIC	A	G		
A_flavus_1041045517023	2477114	WT60-3	33315	G	C	AFL2T_04746	Has domain(s) with predicted mismatched DNA binding, ATP binding activity and role in mismatch repair
A_flavus_1041045517014	127731	WT60-3	INTERGENIC	G	T		
A_flavus_1041045517010	253282	WT60-3	27713	C	G	AFL2T_08286	
A_flavus_1041045517029	1638601	WT60-3	37984	G	T	AFL2T_09264	
A_flavus_1041045517028	348172	WT60-3	36900	C	A	AFL2T_10135	
A_flavus_1041045517023	679763	WT60-4	32692	C	T	AFL2T_04123	Protein of unknown function
A_flavus_1041045517023	730255	WT60-4	INTERGENIC	C	A		
A_flavus_1041045517028	1623708	WT60-4	37319	C	A	AFL2T_10554	Protein of unknown function
A_flavus_1041045517025	1425081	WT60-5	INTERGENIC	C	A		
A_flavus_1041045517012	938566	WT60-5	29066	G	C	AFL2T_01864	
A_flavus_1041045517014	1140416	WT60-5	INTERGENIC	G	C		
A_flavus_1041045517027	2114918	WT60-5	36712	C	G	AFL2T_07180	Protein of unknown function
A_flavus_1041045517027	2336922	WT60-5		A	T		
A_flavus_1041045517026	367425	WT60-5	35734	G	C		
A_flavus_1041045517025	2767254	WT60-6	35038	G	T	AFL2T_01218	
A_flavus_1041045517025	2840336	WT60-6	INTERGENIC	A	ATG		
A_flavus_1041045517009	1832327	WT60-6	27076	C	T	AFL2T_03580	Has domain(s) with predicted substrate-specific transmembrane transporter activity, role in transmembrane transport and integral to membrane localization
A_flavus_1041045517009	2603075	WT60-6	27345	G	A	AFL2T_03849	Has domain(s) with predicted nucleic acid binding, nucleotide binding activity
A_flavus_1041045517027	1221825	WT60-6	36418	C	T	AFL2T_06886	
A_flavus_1041045517028	544138	WT60-6	36970	C	G	AFL2T_10205	Protein of unknown function
A_flavus_1041045517025	3187259	WT60-7	34930	TA	T	AFL2T_01110	Has domain(s) with predicted dihydrolipoyllysine-residue succinyltransferase activity, role in tricarboxylic acid cycle and oxoglutarate dehydrogenase complex localization
A_flavus_1041045517012	2498620	WT60-7	29587	C	A	AFL2T_02385	Ortholog(s) have role in cellular calcium ion homeostasis
A_flavus_1041045517009	95700	WT60-7	26470	G	C	AFL2T_02974	
A_flavus_1041045517022	1114969	WT60-7	INTERGENIC	C	G		
A_flavus_1041045517022	1771086	WT60-7	INTERGENIC	C	G		
A_flavus_1041045517024	1172196	WT60-7	33782	G	A	AFL2T_12317	
A_flavus_1041045517007	2502	WT60-7	26322	T	C	AFL2T_12475	
A_flavus_1041045517025	3368573	WT60-8	INTERGENIC	GA	G		
A_flavus_1041045517012	3723286	WT60-8	INTERGENIC	G	T		
A_flavus_1041045517009	558008	WT60-8	INTERGENIC	T	G		

A_flavus_1041045517023	1351413	WT60-8	32918	C	A	AFL2T_04349	Has domain(s) with predicted nucleic acid binding, ATP binding, ATP-dependent helicase activity
A_flavus_1041045516891	199792	WT60-8	25573	G	A	AFL2T_07324	
A_flavus_1041045517012	3588661	WT60-9	29966	G	C	AFL2T_02764	Protein of unknown function
A_flavus_1041045517009	278237	WT60-9	INTERGENIC	G	T		
A_flavus_1041045517023	1985866	WT60-9	33374	C	A		
A_flavus_1041045516891	461871	WT60-9	INTERGENIC	C	A		
A_flavus_1041045517011	1073518	WT60-9	INTERGENIC	G	A		
A_flavus_1041045517024	538065	WT60-9	33575	C	A	AFL2T_12110	Protein of unknown function
A_flavus_1041045516888	4043	WT60-9	INTERGENIC	C	A		
A_flavus_1041045517009	847121	WT60-10	26976	C	A		
A_flavus_1041045517009	1878203	WT60-10	27089	G	T	AFL2T_03593	Has domain(s) with predicted ATP binding, ATPase activity, coupled to transmembrane movement of substances activity, role in transmembrane transport and integral to membrane localization
A_flavus_1041045517021	677404	WT60-10	31234	G	T	AFL2T_05895	Protein of unknown function
A_flavus_1041045517027	187878	WT60-10	36055	A	G	AFL2T_06523	
A_flavus_1041045516891	121175	WT60-10	25544	C	A	AFL2T_07295	

A. flavus ∆ku70

A_flavus_1041045517012	3599082	KU70-60-1	29972	G	A	AFL2T_02770	Has domain(s) with predicted zinc ion binding activity
A_flavus_1041045517027	1682934	KU70-60-1	36812	G	T		
A_flavus_1041045517029	350917	KU70-60-1	INTERGENIC	T	G		
A_flavus_1041045517028	1610011	KU70-60-1	37314	C	T	AFL2T_10549	
A_flavus_1041045517011	877126	KU70-60-1	28408	C	A	AFL2T_10936	Has domain(s) with predicted oxidoreductase activity and role in oxidation-reduction process
A_flavus_1041045517012	320602	KU70-60-2	INTERGENIC	C	A		
A_flavus_1041045517012	2631421	KU70-60-2	29634	C	G	AFL2T_02432	
A_flavus_1041045517009	345973	KU70-60-2	26554	G	A	AFL2T_03058	Ortholog(s) have role in short-chain fatty acid catabolic process, positive regulation of transcription from RNA polymerase II promoter
A_flavus_1041045517009	483004	KU70-60-2	26608	G	C	AFL2T_03112	Has domain(s) with predicted sequence-specific DNA binding RNA polymerase II transcription factor, zinc ion binding activity, role in regulation of transcription, DNA-dependent and nucleus localization
A_flavus_1041045517023	375963	KU70-60-2	32588	C	T	AFL2T_04019	
A_flavus_1041045517023	1002813	KU70-60-2	INTERGENIC	T	C		
A_flavus_1041045517028	1415691	KU70-60-2	37488	C	A	AFL2T_08768	Has domain(s) with predicted hydrolase activity
A_flavus_1041045517012	1922756	KU70-60-3	29636	C	A	AFL2T_02434	
A_flavus_1041045517012	3451811	KU70-60-3	30158	C	A	AFL2T_04828	Protein of unknown function
A_flavus_1041045517014	401374	KU70-60-3	INTERGENIC	G	A		
A_flavus_1041045516891	829780	KU70-60-3	25796	A	G	AFL2T_07547	
A_flavus_1041045517024	529740	KU70-60-3	33571	C	A	AFL2T_12106	
A_flavus_1041045517012	91029	KU70-60-4	INTERGENIC	G	T		
A_flavus_1041045517023	55808	KU70-60-4	INTERGENIC	AAGAGT	A		
A_flavus_1041045517014	565416	KU70-60-4	30566	G	T		
A_flavus_1041045517014	684117	KU70-60-4	INTERGENIC	T	A		
A_flavus_1041045517026	1156937	KU70-60-4	35989	G	T	AFL2T_10009	Protein of unknown function
A_flavus_1041045517028	1561319	KU70-60-4	37296	C	A	AFL2T_10531	Protein of unknown function
A_flavus_1041045517022	21141	KU70-60-4	31816	TACC	T	AFL2T_11276	
A_flavus_1041045517022	944397	KU70-60-4	32152	C	A	AFL2T_11612	Has domain(s) with predicted 6-phosphofructokinase activity, role in glycolysis, fructose 6-phosphate metabolic process and 6-phosphofructokinase complex localization
A_flavus_1041045517024	487207	KU70-60-4	INTERGENIC	G	C		
A_flavus_1041045517008	151341	KU70-60-4	26369	G	T	AFL2T_12402	Has domain(s) with predicted monooxygenase, iron ion binding, electron carrier, heme binding, oxidoreductase activity, acting on paired donors, with incorporation or reduction of molecular oxygen activity and role in oxidation-reduction process
A_flavus_1041045517025	3167201	KU70-60-5	35165	G	T	AFL2T_01345	
A_flavus_1041045517014	2054988	KU70-60-5	30835	A	C	AFL2T_05505	Has domain(s) with predicted ammonia-lyase activity, role in L-phenylalanine catabolic process, biosynthetic process and cytoplasm localization
A_flavus_1041045517021	1010968	KU70-60-5	INTERGENIC	C	G		
A_flavus_1041045517010	228027	KU70-60-5	27463	C	A	AFL2T_08036	

A_flavus_1041045517029	947132	KU70-60-5	INTERGENIC	G	C		
A_flavus_1041045517029	1610886	KU70-60-5	37972	T	TGCTAG	AFL2T_09252	
A_flavus_1041045517022	310569	KU70-60-5	32158	G	A	AFL2T_11618	Protein of unknown function
A_flavus_1041045517009	222145	KU70-60-6	26511	G	A	AFL2T_03015	
A_flavus_1041045517023	603924	KU70-60-6	INTERGENIC	C	A		
A_flavus_1041045517014	2161421	KU70-60-6	INTERGENIC	TTATCT(	T		
A_flavus_1041045517027	1856902	KU70-60-6	36620	C	T	AFL2T_07088	Has domain(s) with predicted role in transmembrane transport and integral to membrane localization
A_flavus_1041045517010	353040	KU70-60-6	27513	G	T	AFL2T_08086	Has domain(s) with predicted protein kinase, ATP binding activity and role in protein phosphorylation
A_flavus_1041045517029	553424	KU70-60-6	INTERGENIC	G	A		
A_flavus_1041045517029	1381550	KU70-60-6	INTERGENIC	C	G		
A_flavus_1041045517026	115162	KU70-60-6	INTERGENIC	G	C		
A_flavus_1041045517011	1315663	KU70-60-6	28569	G	A	AFL2T_11097	Has domain(s) with predicted intramolecular transferase activity
A_flavus_1041045517022	1092464	KU70-60-6	32202	G	T	AFL2T_11662	
A_flavus_1041045517022	1483554	KU70-60-6	32347	G	T	AFL2T_11807	
A_flavus_1041045517008	152760	KU70-60-6	INTERGENIC	A	ACTC		
A_flavus_1041045517025	2063049	KU70-60-7	34548	G	A	AFL2T_00728	Has domain(s) with predicted hydrolase activity, hydrolyzing O-glycosyl compounds activity and role in carbohydrate metabolic process
A_flavus_1041045517012	4066248	KU70-60-7	INTERGENIC	T	TTC		
A_flavus_1041045517021	1403758	KU70-60-7	INTERGENIC	C	G		
A_flavus_1041045516891	276341	KU70-60-7	INTERGENIC	C	G		
A_flavus_1041045517010	1356580	KU70-60-7	28092	G	A	AFL2T_08665	
A_flavus_1041045517029	13644	KU70-60-7	37404	T	C	AFL2T_08684	
A_flavus_1041045517029	1908930	KU70-60-7	38065	T	G	AFL2T_09345	Protein of unknown function
A_flavus_1041045517026	879488	KU70-60-7	35662	G	GA	AFL2T_09682	
A_flavus_1041045517011	601178	KU70-60-7	INTERGENIC	T	TTG		
A_flavus_1041045517025	2673467	KU70-60-8	34762	G	T	AFL2T_00942	Has domain(s) with predicted flavin adenine dinucleotide binding, oxidoreductase activity, acting on CH-OH group of donors activity and role in oxidation-reduction process
A_flavus_1041045517023	765092	KU70-60-8	INTERGENIC	G	T		
A_flavus_1041045517014	1212683	KU70-60-8	INTERGENIC	G	T		
A_flavus_1041045517014	1597961	KU70-60-8	INTERGENIC	A	C		
A_flavus_1041045517027	2236635	KU70-60-8	INTERGENIC	G	GTAC		
A_flavus_1041045517029	1696696	KU70-60-8	38244	G	T		
A_flavus_1041045517025	2591	KU70-60-9	33821	C	A	AFL2T_00001	Protein of unknown function
A_flavus_1041045517012	1068942	KU70-60-9	29111	C	A	AFL2T_01909	
A_flavus_1041045516891	1238100	KU70-60-9	25940	T	G	AFL2T_07691	Protein of unknown function
A_flavus_1041045517010	199448	KU70-60-9	INTERGENIC	C	A		
A_flavus_1041045517010	793333	KU70-60-9	27664	C	T	AFL2T_08237	Protein of unknown function
A_flavus_1041045517022	2465	KU70-60-9	31809	C	G	AFL2T_11269	Protein of unknown function
A_flavus_1041045517022	917087	KU70-60-9	32143	A	AAC	AFL2T_11603	
A_flavus_1041045517024	1112278	KU70-60-9	34007	C	A	AFL2T_00187	
A_flavus_1041045517025	2183583	KU70-60-10	INTERGENIC	CT	C		
A_flavus_1041045517012	333348	KU70-60-10	INTERGENIC	G	T		
A_flavus_1041045517014	1781398	KU70-60-10	30980	G	T	AFL2T_05650	Has domain(s) with predicted protein binding, phosphatidylinositol binding, DNA binding, transcription coactivator activity and role in cell communication, regulation of transcription, DNA-dependent
A_flavus_1041045516891	1758236	KU70-60-10	26109	G	T	AFL2T_07860	Protein of unknown function
A_flavus_1041045517010	1669303	KU70-60-10	INTERGENIC	T	G		
A_flavus_1041045517028	1611559	KU70-60-10	37557	G	C	AFL2T_08837	
A_flavus_1041045517011	1602947	KU70-60-10	INTERGENIC	A	AGT		
