# Supplementary material for: Genome sequencing of evolved aspergilli populations reveals robust genomes, transversions in A. flavus, and sexual aberrancy in non-homologous end-joining mutants
Source: BMC Biol. 2019 Nov 11;17:88. doi: 10.1186/s12915-019-0702-0 (PMC6844060; doi:10.1186/s12915-019-0702-0)
Supplement: Supplementary file 6 — Additional file 6: Table S2. Genes mutated during the MA experiment in A. fumigatus. [file 12915_2019_702_MOESM6_ESM.pdf]

STRAIN AND CHROMOSOME	POSITION	MA LINE	GENE	REF	ALT	ANOTATION
***A. fumigatus* WT**						
Chr_1_A_fumigatus_Af293	1906334	WT60-1	Afu1g06660	G	GC	Protein of unknown function
Chr_2_A_fumigatus_Af293	2215206	WT60-1	Afu2g08640	G	A	Ortholog of A. nidulans FGSC A4 : AN6137, A. niger CBS 513.88 : An12g03990, Aspergillus wentii : Aspwe1_0176126, Aspergillus sydowii : Aspsy1_0058040 and Aspergillus terreus NIH2624 : ATET_09699
Chr_1_A_fumigatus_Af293	4238270	WT60-2	Afu1g15690	T	G	Ortholog of A. nidulans FGSC A4 : AN0903, A. niger CBS 513.88 : An01g14030, A. oryzae RIB40 : AO090005001138, Aspergillus wentii : Aspwe1_0027550 and Aspergillus sydowii : Aspsy1_0139000
Chr_1_A_fumigatus_Af293	3495339	WT60-3	Afu1g13170	A	G	Ortholog(s) have role in septation initiation signaling and mitotic spindle pole body localization
Chr_2_A_fumigatus_Af293	2471087	WT60-3	INTERGENIC	A	G	
Chr_2_A_fumigatus_Af293	2781396	WT60-3	Afu2g10830	T	C	Ortholog of Aspergillus fumigatus A1163 : AFUB_026610
Chr_4_A_fumigatus_Af293	3574910	WT60-3	Afu4g13660	T	C	Ortholog(s) have drug transmembrane transporter activity, polyamine transmembrane transporter activity and role in ascospore wall assembly, drug transmembrane transport, lipid homeostasis, membrane raft assembly, polyamine homeostasis
Chr_6_A_fumigatus_Af293	840745	WT60-3	Afu6g03820	C	CCGTGGT	Nascent polypeptide-associated complex subunit alpha; predicted role in 'de novo' cotranslational protein folding; enriched in dormant conidia
Chr_3_A_fumigatus_Af293	2009857	WT60-4	INTERGENIC	G	A	
Chr_6_A_fumigatus_Af293	3377779	WT60-5	Afu6g13310	T	C	Ortholog(s) have role in proteasome regulatory particle assembly and cytosol, nucleus localization
Chr_1_A_fumigatus_Af293	2324518	WT60-9	Afu1g08960	C	A	Ortholog of A. nidulans FGSC A4 : AN1383, A. niger CBS 513.88 : An08g00450, A. oryzae RIB40 : AO090005001630, Aspergillus wentii : Aspwe1_0048794 and Aspergillus sydowii : Aspsy1_0025360
Chr_6_A_fumigatus_Af293	1720540	WT60-10	Afu6g07570	C	A	Has domain(s) with predicted zinc ion binding activity
A. fumigatus ∆ku70						
Chr_2_A_fumigatus_Af293	1479836	KU70-60-1	INTERGENIC	T	C	
Chr_8_A_fumigatus_Af293	690852	KU70-60-1	INTERGENIC	A	G	
Chr_4_A_fumigatus_Af293	2718238	KU70-60-2	INTERGENIC	C	CTCATCA	
Chr_5_A_fumigatus_Af293	550266	KU70-60-2	Afu5g02160	AGGGG	A	Has domain(s) with predicted exonuclease activity, nucleic acid binding, nucleotide binding, zinc ion binding activity and intracellular localization
Chr_6_A_fumigatus_Af293	2637054	KU70-60-3	Afu6g10660	G	GATGCACG	Putative ATP citrate lyase subunit; repressed by gliotoxin exposure22405895
Chr_1_A_fumigatus_Af293	680900	KU70-60-4	INTERGENIC	T	C	
Chr_3_A_fumigatus_Af293	912520	KU70-60-5	Afu3g03420	T	A	Fusarinine C non-ribosomal peptide synthetase (NRPS) involved in extracellular siderophore biosynthesis; expression is up-regulated by iron starvation and downregulated under iron-replete conditions
Chr_2_A_fumigatus_Af293	2102173	KU70-60-6	Afu2g08100	T	TGTCCTGA	Ortholog of Aspergillus fumigatus A1163 : AFUB_024119
Chr_2_A_fumigatus_Af293	3171393	KU70-60-6	Afu2g12340	C	A	Has domain(s) with predicted role in meiotic cell cycle
Chr_6_A_fumigatus_Af293	378799	KU70-60-6	Afu6g02170	A	G	Ortholog(s) have ferric-chelate reductase activity, role in copper ion import, iron ion transport and plasma membrane localization
Chr_6_A_fumigatus_Af293	1014987	KU70-60-6	Afu6g04440	A	G	Ortholog(s) have role in mitochondrial tRNA wobble uridine modification and mitochondrion localization
Chr_3_A_fumigatus_Af293	111746	KU70-60-7	Afu3g00510	G	A	Ortholog of Aspergillus fumigatus A1163 : AFUB_047940
Chr_3_A_fumigatus_Af293	1619358	KU70-60-7	INTERGENIC	A	ATTC	
Chr_4_A_fumigatus_Af293	3368853	KU70-60-7	Afu4g12870	C	T	Putative methylmalonate-semialdehyde dehydrogenase; transcript up-regulated in conidia exposed to neutrophils
Chr_6_A_fumigatus_Af293	3105415	KU70-60-7	Afu6g12340	C	T	Putative GTPase-activating protein; transcript upregulated in response to amphotericin B
Chr_3_A_fumigatus_Af293	3242180	KU70-60-8	INTERGENIC	CCT	C	
Chr_3_A_fumigatus_Af293	3242184	KU70-60-8	INTERGENIC	A	AAG	
Chr_4_A_fumigatus_Af293	1130472	KU70-60-8	Afu4g04010	G	C	Ortholog(s) have role in fatty acid catabolic process
Chr_6_A_fumigatus_Af293	3240470	KU70-60-8	INTERGENIC	A	G	
Chr_4_A_fumigatus_Af293	3667426	KU70-60-9	INTERGENIC	G	A	
Chr_6_A_fumigatus_Af293	851014	KU70-60-9	Afu6g03870	G	A	Protein of unknown function; transcript downregulated in response to voriconazole
Chr_3_A_fumigatus_Af293	508823	KU70-60-10	Afu3g02070	C	T	Has domain(s) with predicted DNA binding, nucleic acid binding, zinc ion binding activity, role in transcription, DNA-templated and nucleus localization
Chr_4_A_fumigatus_Af293	2484127	KU70-60-10	Afu4g09490	C	T	Ortholog of Aspergillus fumigatus A1163 : AFUB_066610
Chr_7_A_fumigatus_Af293	709420	KU70-60-10	Afu7g02580	C	G	Predicted DDE1 transposon-related ORF; transcript induced by exposure to human airway epithelial cells
Chr_8_A_fumigatus_Af293	678707	KU70-60-10	INTERGENIC	TAC	T	
