# Supplementary material for: Genome sequencing of evolved aspergilli populations reveals robust genomes, transversions in A. flavus, and sexual aberrancy in non-homologous end-joining mutants
Source: BMC Biol. 2019 Nov 11;17:88. doi: 10.1186/s12915-019-0702-0 (PMC6844060; doi:10.1186/s12915-019-0702-0)
Supplement: Supplementary file 7 — Additional file 7: Table S3. Genes mutated during the asexual MA experiment in A. nidulans. [file 12915_2019_702_MOESM7_ESM.pdf]

STRAIN AND CHROMOSOME	POSITION	MA LINE	GENE	REF	ALT	ANOTATION
***A. nidulans* WT - Asexual MA lines**						
ChrIV_A_nidulans_FGSC_A4	483735	ASEXUAL_WT_60_1	AN7269	G	A	Ortholog(s) have role in fumiquinazoline C biosynthetic process, secondary metabolite biosynthetic process and fungal-type cell wall localization
ChrIV_A_nidulans_FGSC_A4	547255	ASEXUAL_WT_60_1	AN7248	T	A	Ortholog of A. fumigatus Af293 : Afu2g17200, A. niger CBS 513.88 : An04g09270, A. oryzae RIB40 : AO090102000099, Aspergillus wentii : Aspwe1_0153703 and Aspergillus sydowii : Aspsy1_0140330
ChrIV_A_nidulans_FGSC_A4	1520349	ASEXUAL_WT_60_1	INTERGENIC	C	CT	
ChrI_A_nidulans_FGSC_A4	1010020	ASEXUAL_WT_60_1	INTERGENIC	A	AAC	
ChrVIII_A_nidulans_FGSC_A4	2169263	ASEXUAL_WT_60_1	INTERGENIC	C	CA	
ChrVII_A_nidulans_FGSC_A4	3059130	ASEXUAL_WT_60_1	AN2157	A	G	Putative aspartic endopeptidase
ChrV_A_nidulans_FGSC_A4	1392706	ASEXUAL_WT_60_1	AN5173	T	TTG	Has domain(s) with predicted hydrolase activity
ChrVIII_A_nidulans_FGSC_A4	2155552	ASEXUAL_WT_60_2	AN0890	A	G	Ortholog(s) have fungal-type vacuole membrane localization
ChrVIII_A_nidulans_FGSC_A4	4010892	ASEXUAL_WT_60_2	INTERGENIC	CTGGTAGG	C	
ChrIII_A_nidulans_FGSC_A4	1010863	ASEXUAL_WT_60_3	AN4783	G	T	Subunit 2 of the COP9 signalosome; required for formation of cleistothecia; mutants produce aberrant red color within distinct hyphae
ChrIV_A_nidulans_FGSC_A4	341689	ASEXUAL_WT_60_3	AN7311	C	T	Ortholog of A. fumigatus Af293 : Afu2g16670, A. niger CBS 513.88 : An04g08690, A. oryzae RIB40 : AO090102000156, Aspergillus wentii : Aspwe1_0039317 and Aspergillus sydowii : Aspsy1_0027138
ChrVII_A_nidulans_FGSC_A4	1960358	ASEXUAL_WT_60_3	AN1810	C	T	Ornithine transaminase, involved in utilization of arginine as a proline source; arginine-induced expression and activity; subject to carbon-catabolite and nitrogen-metabolite repression; regulated by CreA and AreA; stress-induced protein
ChrVI_A_nidulans_FGSC_A4	312083	ASEXUAL_WT_60_3	INTERGENIC	C	G	
ChrVI_A_nidulans_FGSC_A4	1747837	ASEXUAL_WT_60_3	INTERGENIC	G	A	
ChrIII_A_nidulans_FGSC_A4	1151464	ASEXUAL_WT_60_5	AN4733	G	GGTT	Ortholog(s) have role in signal transduction and plasma membrane localization
ChrII_A_nidulans_FGSC_A4	182082	ASEXUAL_WT_60_5	INTERGENIC	G	A	
ChrII_A_nidulans_FGSC_A4	992968	ASEXUAL_WT_60_5	AN8168	C	T	Regulatory protein involved in nitrogen metabolite repression
ChrII_A_nidulans_FGSC_A4	2977222	ASEXUAL_WT_60_5	AN3814	CCTTCT	C	Putative peptidyl-prolyl cis-trans isomerase (PPIase); cyclophilin
ChrVIII_A_nidulans_FGSC_A4	219652	ASEXUAL_WT_60_5	INTERGENIC	AT	A	
ChrVIII_A_nidulans_FGSC_A4	766114	ASEXUAL_WT_60_5	INTERGENIC	G	A	
ChrVIII_A_nidulans_FGSC_A4	2996437	ASEXUAL_WT_60_5	AN0609	C	T	Triacetylfusarinine C (TAFC) biosynthetic enoyl-CoA hydratase; siderophore biosynthetic enzyme
ChrVI_A_nidulans_FGSC_A4	977075	ASEXUAL_WT_60_5	INTERGENIC	G	GCCAAGGAAT	
ChrV_A_nidulans_FGSC_A4	552192	ASEXUAL_WT_60_5	INTERGENIC	T	C	
ChrIII_A_nidulans_FGSC_A4	2574750	ASEXUAL_WT_60_6	AN8860	G	A	Ortholog(s) have ATP binding, ATPase activity, adenylate kinase activity
ChrII_A_nidulans_FGSC_A4	3185314	ASEXUAL_WT_60_6	AN3750	C	A	Ortholog of A. fumigatus Af293 : Afu7g04470, A. niger CBS 513.88 : An13g01050, A. oryzae RIB40 : AO090005000133, Aspergillus wentii : Aspwe1_0038525 and Aspergillus sydowii : Aspsy1_0088662
ChrI_A_nidulans_FGSC_A4	128344	ASEXUAL_WT_60_6	AN6452	C	G	Ortholog(s) have oligopeptide transmembrane transporter activity and role in transmembrane transport
ChrI_A_nidulans_FGSC_A4	3519129	ASEXUAL_WT_60_6	AN6901	G	C	Protein required for wild-type mitochondrial morphology and intracellular mitochondrial distribution; localized to mitochondrial membranes; 4 transmembrane motifs; similar to S. cerevisiae Mdm10p
ChrVIII_A_nidulans_FGSC_A4	2455893	ASEXUAL_WT_60_6	AN0795	C	T	Ortholog of A. fumigatus Af293 : Afu1g14590, A. niger CBS 513.88 : An01g12590, Neosartorya fischeri NRRL 181 : NFIA_010830, Aspergillus wentii : Aspwe1_0027661 and Aspergillus versicolor : Aspve1_0121944
ChrIII_A_nidulans_FGSC_A4	1061875	ASEXUAL_WT_60_8	AN4766	CGCATCTG	C	Ortholog of A. fumigatus Af293 : Afu3g06495, A. niger CBS 513.88 : An11g09830, Aspergillus wentii : Aspwe1_0029358, Aspergillus sydowii : Aspsy1_0087849 and Aspergillus terreus NIH2624 : ATET_08768
ChrIV_A_nidulans_FGSC_A4	1708284	ASEXUAL_WT_60_8	INTERGENIC	G	GTGTTGT	
ChrIV_A_nidulans_FGSC_A4	2478363	ASEXUAL_WT_60_8	AN7721	T	C	Putative translocon, alpha subunit; ortholog of S. cerevisiae Sec61p
ChrVIII_A_nidulans_FGSC_A4	1493647	ASEXUAL_WT_60_8	AN1104	C	A	Ortholog(s) have ADP binding, peptidase activity, role in protein targeting to mitochondrion and mitochondrial processing peptidase complex localization
ChrVI_A_nidulans_FGSC_A4	1436309	ASEXUAL_WT_60_8	INTERGENIC	TGGGGGGG	T	
ChrVI_A_nidulans_FGSC_A4	1659321	ASEXUAL_WT_60_8	INTERGENIC	GC	G	
ChrIII_A_nidulans_FGSC_A4	259683	ASEXUAL_WT_60_10	AN5021	G	T	Ortholog(s) have role in conidium formation, regulation of growth rate
ChrII_A_nidulans_FGSC_A4	3411660	ASEXUAL_WT_60_10	INTERGENIC	T	TGGAAAGGAG	
ChrVIII_A_nidulans_FGSC_A4	2625099	ASEXUAL_WT_60_10	INTERGENIC	G	A	
ChrVIII_A_nidulans_FGSC_A4	2921590	ASEXUAL_WT_60_10	AN0640	G	T	Protein with a role in sphingoglycolipid metabolism; required for phytosphingosine biosynthesis; depletion causes cell wall thickening, reduced asexual development, increased sexual development, increased levels of ppoA and steA transcript
ChrVIII_A_nidulans_FGSC_A4	4035404	ASEXUAL_WT_60_10	AN0283	AGAG	A	Protein of unknown function
ChrVIII_A_nidulans_FGSC_A4	4804512	ASEXUAL_WT_60_10	INTERGENIC	C	T	
ChrVII_A_nidulans_FGSC_A4	3651303	ASEXUAL_WT_60_10	INTERGENIC	G	A	
ChrIII_A_nidulans_FGSC_A4	75776	ASEXUAL_WT_60_4	INTERGENIC	C	T	
ChrI_A_nidulans_FGSC_A4	1414631	ASEXUAL_WT_60_4	AN12016	A	AC	Protein of unknown function
ChrVIII_A_nidulans_FGSC_A4	1328152	ASEXUAL_WT_60_4	INTERGENIC	G	GA	
ChrIV_A_nidulans_FGSC_A4	1112300	ASEXUAL_WT_60_9	INTERGENIC	T	G	
ChrVIII_A_nidulans_FGSC_A4	87223	ASEXUAL_WT_60_7	AN9259	C	T	Predicted prenyltransferase; required for austinol and dehydroaustinol biosynthesis; aus secondary metabolism gene cluster member
ChrVIII_A_nidulans_FGSC_A4	3031041	ASEXUAL_WT_60_7	INTERGENIC	A	AT	

ChrVIII_A_nidulans_FGSC_A4	3438253	ASEXUAL_WT_60_7	AN0477	G	A	Has domain(s) with predicted homogentisate 1,2-dioxygenase activity and role in L-phenylalanine catabolic process, oxidation-reduction process, tyrosine metabolic process
ChrVIII_A_nidulans_FGSC_A4	4331060	ASEXUAL_WT_60_7	AN0191	C	G	Ortholog(s) have ATPase activity, tRNA binding activity and role in regulation of cytoplasmic translational fidelity, regulation of transcription by RNA polymerase II, tRNA wobble base 5-methoxycarbonylmethyl-2-thiouridinylation
ChrVII_A_nidulans_FGSC_A4	2072100	ASEXUAL_WT_60_7	AN1854	C	T	Putative inositol pentakisphosphate 2-kinase; locus contains the conserved upstream open reading frame (uORF) AN1854-uORF

***A. nidulans ∆ku70* - Asexual MA lines**

ChrII_A_nidulans_FGSC_A4	439614	ASEXUAL_KU70_60_1	INTERGENIC	G	GT	
ChrII_A_nidulans_FGSC_A4	1909882	ASEXUAL_KU70_60_1	INTERGENIC	C	T	
ChrII_A_nidulans_FGSC_A4	2446569	ASEXUAL_KU70_60_1	AN10502	G	T	Has domain(s) with predicted catalytic activity, hydrolase activity, hydrolyzing O-glycosyl compounds activity and role in carbohydrate metabolic process
ChrII_A_nidulans_FGSC_A4	3438377	ASEXUAL_KU70_60_1	INTERGENIC	C	T	
ChrI_A_nidulans_FGSC_A4	2943315	ASEXUAL_KU70_60_1	AN6712	C	A	Putative phospholipase D; expression upregulated after exposure to farnesol
ChrVIII_A_nidulans_FGSC_A4	524147	ASEXUAL_KU70_60_1	AN9402	T	G	Has domain(s) with predicted hydrolase activity and role in nucleotide catabolic process
ChrVIII_A_nidulans_FGSC_A4	3212384	ASEXUAL_KU70_60_1	AN0549	C	A	Putative chitinase; glycoside hydrolase family 18 (GH18) protein with a predicted role in chitin hydrolysis
ChrVII_A_nidulans_FGSC_A4	43723	ASEXUAL_KU70_60_1	AN9047	G	T	Has domain(s) with predicted DNA binding, RNA polymerase II transcription factor activity, sequence-specific DNA binding, zinc ion binding activity and role in regulation of transcription, DNA-templated, transcription, DNA-templated
ChrVII_A_nidulans_FGSC_A4	4395435	ASEXUAL_KU70_60_1	INTERGENIC	G	A	
ChrVI_A_nidulans_FGSC_A4	1174429	ASEXUAL_KU70_60_1	AN3307	C	T	Catalytic subunit of the major alpha-1,3 glucan synthase complex; mutants grow as dispersed hyphae in liquid culture
ChrVI_A_nidulans_FGSC_A4	2423143	ASEXUAL_KU70_60_1	INTERGENIC	C	A	
ChrII_A_nidulans_FGSC_A4	3069809	ASEXUAL_KU70_60_2	AN3783	G	T	Protein of unknown function
ChrIV_A_nidulans_FGSC_A4	64187	ASEXUAL_KU70_60_2	AN7402	G	T	Putative glucoamylase with a predicted role in starch metabolism
ChrI_A_nidulans_FGSC_A4	175610	ASEXUAL_KU70_60_2	AN6439	A	G	Ortholog(s) have intracellular localization
ChrVIII_A_nidulans_FGSC_A4	1158569	ASEXUAL_KU70_60_2	INTERGENIC	A	G	
ChrVIII_A_nidulans_FGSC_A4	4459842	ASEXUAL_KU70_60_2	AN10021	C	T	Regulatory protein; member of the monodictyphenone secondary metabolite biosynthesis gene cluster; similar to aflatoxin coactivator aflJ and O-methyltransferases; required for biosynthesis of monodictyphenone, a prenyl xanthone precursor
ChrVII_A_nidulans_FGSC_A4	3184055	ASEXUAL_KU70_60_2	INTERGENIC	C	CA	
ChrIII_A_nidulans_FGSC_A4	145032	ASEXUAL_KU70_60_6	INTERGENIC	A	G	
ChrIV_A_nidulans_FGSC_A4	122691	ASEXUAL_KU70_60_6	INTERGENIC	T	A	
ChrIV_A_nidulans_FGSC_A4	2801413	ASEXUAL_KU70_60_6	INTERGENIC	C	G	
ChrIV_A_nidulans_FGSC_A4	2801416	ASEXUAL_KU70_60_6	INTERGENIC	C	G	
ChrVIII_A_nidulans_FGSC_A4	4448922	ASEXUAL_KU70_60_6	AN0150	G	A	Polyketide synthase; member of the monodictyphenone secondary metabolite biosynthesis gene cluster; required for the synthesis of the xanthones (shamixanthone and epishamixanthone), emodin, arugosins, monodictyphenone, and related compounds
ChrVI_A_nidulans_FGSC_A4	3147687	ASEXUAL_KU70_60_6	AN2686	C	T	Has domain(s) with predicted role in mitotic spindle organization and DASH complex, mitotic spindle localization
ChrV_A_nidulans_FGSC_A4	2862607	ASEXUAL_KU70_60_6	INTERGENIC	T	TA	
ChrIII_A_nidulans_FGSC_A4	1900834	ASEXUAL_KU70_60_7	AN4483	C	A	Predicted protein serine/threonine kinase, part of HogA mitogen-activated protein kinase (MAPK) signaling pathway, involved in regulation of stress response and development
ChrII_A_nidulans_FGSC_A4	3019588	ASEXUAL_KU70_60_7	AN3797	C	G	Ortholog(s) have role in cellular response to UV, double-strand break repair via homologous recombination, postreplication repair
ChrI_A_nidulans_FGSC_A4	2384215	ASEXUAL_KU70_60_7	INTERGENIC	GTTT	G	
ChrVI_A_nidulans_FGSC_A4	2044416	ASEXUAL_KU70_60_7	INTERGENIC	T	G	
ChrII_A_nidulans_FGSC_A4	250784	ASEXUAL_KU70_60_9	INTERGENIC	C	T	
ChrVII_A_nidulans_FGSC_A4	2508300	ASEXUAL_KU70_60_9	INTERGENIC	T	G	
ChrVII_A_nidulans_FGSC_A4	3649095	ASEXUAL_KU70_60_9	AN2349	C	A	Putative ATP-binding cassette (ABC) transporter of the P-glycoprotein cluster
ChrVI_A_nidulans_FGSC_A4	2713410	ASEXUAL_KU70_60_9	AN2818	A	G	Ortholog of A. niger CBS 513.88 : An12g03370, An18g00460, An07g00580, A. oryzae RIB40 : AO090103000478, Aspergillus wentii : Aspwe1_0058229, Aspwe1_0111514 and Aspergillus versicolor : Aspve1_0051884
ChrV_A_nidulans_FGSC_A4	333035	ASEXUAL_KU70_60_9	AN8388	T	TA	Has domain(s) with predicted catalytic activity and role in nucleoside metabolic process
ChrIII_A_nidulans_FGSC_A4	619577	ASEXUAL_KU70_60_10	AN4900	C	A	Putative bZIP DNA-binding protein involved in regulating nitrogen metabolite repression
ChrIII_A_nidulans_FGSC_A4	840657	ASEXUAL_KU70_60_10	AN4835	G	A	Has domain(s) with predicted role in cell division, centromere complex assembly, chromosome segregation and kinetochore, nucleus localization
ChrIII_A_nidulans_FGSC_A4	2321136	ASEXUAL_KU70_60_10	AN4349	T	C	Ortholog of A. fumigatus Af293 : Afu4g06480, A. niger CBS 513.88 : An04g00800, A. oryzae RIB40 : AO090023000945, Aspergillus wentii : Aspwe1_0100642 and Aspergillus sydowii : Aspsy1_0163308
ChrIII_A_nidulans_FGSC_A4	3421031	ASEXUAL_KU70_60_10	INTERGENIC	G	C	
ChrII_A_nidulans_FGSC_A4	1789495	ASEXUAL_KU70_60_10	INTERGENIC	G	C	
ChrIV_A_nidulans_FGSC_A4	2756239	ASEXUAL_KU70_60_10	AN7814	G	A	Putative polyketide synthase/fatty acid synthase beta; required for sterigmatocystin biosynthesis; member of the sterigmatocystin biosynthesis gene cluster
ChrVIII_A_nidulans_FGSC_A4	3612368	ASEXUAL_KU70_60_10	INTERGENIC	T	A	
ChrVI_A_nidulans_FGSC_A4	117320	ASEXUAL_KU70_60_10	INTERGENIC	G	GTATACTTGCCTACAACT	
ChrVI_A_nidulans_FGSC_A4	1828305	ASEXUAL_KU70_60_10	INTERGENIC	A	G	
ChrIII_A_nidulans_FGSC_A4	982379	ASEXUAL_KU70_60_3	INTERGENIC	C	T	
ChrI_A_nidulans_FGSC_A4	736551	ASEXUAL_KU70_60_3	INTERGENIC	T	TTG	
ChrI_A_nidulans_FGSC_A4	1991031	ASEXUAL_KU70_60_3	INTERGENIC	A	G	

ChrVIII_A_nidulans_FGSC_A4	1389464	ASEXUAL_KU70_60_3	INTERGENIC	C	CTTTTTTTTCT	
ChrVII_A_nidulans_FGSC_A4	930902	ASEXUAL_KU70_60_3	AN10195	C	T	Ortholog(s) have valine-tRNA ligase activity
ChrII_A_nidulans_FGSC_A4	674085	ASEXUAL_KU70_60_5	AN8076	G	GCAC	Predicted DNA binding protein; locus contains the conserved upstream open reading frame (uORF) AN8076-uORF
ChrVI_A_nidulans_FGSC_A4	9979	ASEXUAL_KU70_60_5	AN9233	G	T	Predicted O-methyltransferase; member of a dimethyl-allyl-tryptophan synthase (DMATS) type aromatic prenyltransferase- and NRPS-containing gene cluster
ChrVI_A_nidulans_FGSC_A4	2865880	ASEXUAL_KU70_60_5	AN10340	G	C	Ortholog(s) have di-trans,poly-cis-decaprenylcistransferase activity, trans-hexaprenyltranstransferase activity and role in farnesyl diphosphate biosynthetic process, mevalonate pathway, ubiquinone biosynthetic process
ChrVII_A_nidulans_FGSC_A4	3216198	ASEXUAL_KU70_60_8	INTERGENIC	TTGCCTCAC	T	
ChrVII_A_nidulans_FGSC_A4	3287940	ASEXUAL_KU70_60_8	INTERGENIC	C	CTCTTTTCTTT	
ChrVII_A_nidulans_FGSC_A4	3287957	ASEXUAL_KU70_60_8	INTERGENIC	T	C	
ChrVI_A_nidulans_FGSC_A4	2200530	ASEXUAL_KU70_60_8	INTERGENIC	A	G	
