# Supplementary material for: Genome sequencing of evolved aspergilli populations reveals robust genomes, transversions in A. flavus, and sexual aberrancy in non-homologous end-joining mutants
Source: BMC Biol. 2019 Nov 11;17:88. doi: 10.1186/s12915-019-0702-0 (PMC6844060; doi:10.1186/s12915-019-0702-0)
Supplement: Supplementary file 8 — Additional file 8: Table S4 Genes mutated during the sexual MA experiment in A. nidulans. [file 12915_2019_702_MOESM8_ESM.pdf]

STRAIN AND CHROMOSOME	POSITION	MA LINE	GENE	REF	ALT	ANOTATION
***A. nidulans* WT - Sexual MA lines**						
ChrIII_A_nidulans_FGSC_A4	3260650	SEXUAL_WT_5_1	INTERGENIC	A	T	
ChrI_A_nidulans_FGSC_A4	1296224	SEXUAL_WT_5_2	AN6089	G	C	Putative 60 kilodalton heat shock protein
ChrVII_A_nidulans_FGSC_A4	3174103	SEXUAL_WT_5_2	INTERGENIC	C	CT	
ChrIV_A_nidulans_FGSC_A4	1124655	SEXUAL_WT_5_3	AN7071	T	C	Putative polyketide synthase; involved in the production of alternariol and other secondary metabolites; predicted backbone enzyme of a secondary metabolism gene cluster
ChrVIII_A_nidulans_FGSC_A4	4403705	SEXUAL_WT_5_9	AN0165	G	GGAA	Ortholog(s) have role in Golgi to vacuole transport, protein targeting to vacuole and AP-3 adaptor complex localization
***A. nidulans* ∆*ku70* - Sexual MA lines**						
ChrII_A_nidulans_FGSC_A4	112335	SEXUAL_KU70_5_2	AN11028	G	A	Predicted nucleotide binding protein with oxidoreductase activity and a role in oxidation-reduction; predicted secondary metabolism gene cluster member
ChrVI_A_nidulans_FGSC_A4	1706688	SEXUAL_KU70_5_2	AN3140	C	A	Ortholog of A. niger CBS 513.88 : An02g08990, A. oryzae RIB40 : AO090012000820, Aspergillus wentii : Aspwe1_0050036, Aspergillus sydowii : Aspsy1_1171750 and Aspergillus terreus NIH2624 : ATET_04095
ChrVIII_A_nidulans_FGSC_A4	66722	SEXUAL_KU70_5_3	INTERGENIC	C	T	
ChrVII_A_nidulans_FGSC_A4	1550386	SEXUAL_KU70_5_3	INTERGENIC	C	T	
ChrVI_A_nidulans_FGSC_A4	1072246	SEXUAL_KU70_5_3	AN3333	T	C	Has domain(s) with predicted oxidoreductase activity
ChrVI_A_nidulans_FGSC_A4	3339468	SEXUAL_KU70_5_3	INTERGENIC	T	A	
ChrIII_A_nidulans_FGSC_A4	2316317	SEXUAL_KU70_5_5	AN10544	G	A	Has domain(s) with predicted 3-dehydroquinate dehydratase activity, catalytic activity
ChrIV_A_nidulans_FGSC_A4	894117	SEXUAL_KU70_5_5	AN10900	C	A	Has domain(s) with predicted serine-type peptidase activity and role in proteolysis
ChrIV_A_nidulans_FGSC_A4	1643084	SEXUAL_KU70_5_5	AN7472	T	C	Ortholog(s) have dolichyl-diphosphooligosaccharide-protein glycotransferase activity, role in protein N-linked glycosylation via asparagine and oligosaccharyltransferase complex, plasma membrane localization
ChrI_A_nidulans_FGSC_A4	2302489	SEXUAL_KU70_5_5	INTERGENIC	A	G	
ChrV_A_nidulans_FGSC_A4	2696819	SEXUAL_KU70_5_5	INTERGENIC	C	T	
ChrV_A_nidulans_FGSC_A4	2696901	SEXUAL_KU70_5_5	INTERGENIC	T	C	
ChrVII_A_nidulans_FGSC_A4	3475139	SEXUAL_KU70_5_7	AN2296	T	C	Ortholog(s) have transcriptional activator activity, RNA polymerase II proximal promoter sequence-specific DNA binding activity, role in positive regulation of transcription by RNA polymerase II and nuclear chromatin localization
ChrII_A_nidulans_FGSC_A4	7382	SEXUAL_KU70_5_8	AN7850	C	A	Ortholog of A. fumigatus Af293 : Afu4g14030, A. oryzae RIB40 : AO090001000391, Neosartorya fischeri NRRL 181 : NFIA_102290, Aspergillus wentii : Aspwe1_0046564 and Aspergillus terreus NIH2624 : ATET_02187
ChrVIII_A_nidulans_FGSC_A4	3910347	SEXUAL_KU70_5_8	INTERGENIC	C	T	
