# Supplementary material for: Genome sequencing of evolved aspergilli populations reveals robust genomes, transversions in A. flavus, and sexual aberrancy in non-homologous end-joining mutants
Source: BMC Biol. 2019 Nov 11;17:88. doi: 10.1186/s12915-019-0702-0 (PMC6844060; doi:10.1186/s12915-019-0702-0)
Supplement: Supplementary file 9 — Additional file 9: Table S5. Strains used in this study. [file 12915_2019_702_MOESM9_ESM.docx]

**Supplementary Table 5:** Strains used in this study

| Strain | Genotype | Reference |
| --- | --- | --- |
| *A. flavus* NRRL3557 | Wild type | FGSC^1^ |
| *A. flavus* TJW149.27 | *∆ku70::pyrG^A. parasiticus^* (derivative of NRRL3557) | ([1](#_ENREF_1)) |
| *A. fumigatus* D141 | Wild type | FGSC |
| *A. fumigatus* AfS35 | *∆akuA::lox-P* (derivative of D141) | ([2](#_ENREF_2)) |
| *A. nidulans* A713 | *yA*2 *pyroA*4; *niiA*4; *veA*1 | FGSC |
| *A. nidulans* A1146 | *wA*3; *pyroA*4; *argB*2; *ΔnkuA*::*argB*; *veA*1 | ([3](#_ENREF_3)) |

^1^ **Fungal Genetic Stock Center** (http://www.fgsc.net/asperg.html)
